# Coupled-mode separation of multiply scattered wavefield components in two-dimensional waveguides

**DOI:** 10.1098/rsos.230352

**Published:** 2023-10-04

**Authors:** Sven M. Ivansson

**Affiliations:** Stockholm University, Stockholm 11529, Sweden

**Keywords:** wavefield decomposition, reflection-matrix recursion, medium truncation

## Abstract

For a waveguide that is invariant in one of the horizontal directions, this paper presents a mathematically exact partial-wave decomposition of the wavefield for assessment of multiple scattering by horizontally displaced medium anomalies. The decomposition is based on discrete coupled-mode theory and combination of reflection/transmission matrices. In particular, there is no high-frequency ray approximation. Full details are presented for the scalar case with plane-wave incidence from below. An application of interest concerns ground motion induced by seismic waves, which may be severely amplified by local medium anomalies such as alluvial valleys. Global optimization techniques are used to design an artificial medium termination at depth for a normal-mode representation of the field. A derivation of a horizontal source array to produce an incident plane wave gives, as a by-product, an extension of a previous Fourier-transform relation involving Bessel functions. Like purely numerical methods, such as finite differences and finite elements, the method can handle all kinds of (two-dimensional) anomaly shapes.

## 1. Introduction

As a complement to purely numerical methods, analytical and semi-analytical techniques are of interest for giving physical insight and for providing bench-mark solutions to wave-propagation problems. This paper presents a semi-analytical method that can separate multiply scattered wavefield components and aid physical interpretation in connection with laterally displaced medium anomalies in a 2D (two-dimensional) waveguide. A discrete coupled-mode method, based on local modes and modal reflection matrices in a medium that is invariant in one of the horizontal directions, is developed for this purpose. The method has the same flexibility concerning anomaly shapes as purely numerical methods. With an artificial medium termination at depth, the wavefield is expanded in terms of normal modes in each of a number of laterally homogeneous strip regions with vertical boundaries. For simplicity, full details and examples are only given for the scalar case with plane-wave incidence from below.

The coupled-mode method has been applied to wave-propagation problems in various disciplines such as acoustics, seismology, electromagnetics, etc.; see [1] for a brief review. For definiteness, seismic waves are considered in the present paper. The discrete variant of the method, with local modes, i.e. modes adapted to the local structure, has been common in underwater acoustics for a long time [2–5]. It is easy to implement numerically using matrix algebra to solve a two-point boundary-value problem for modal expansion coefficients. For the related continuous variant, without discretization of the medium into laterally homogeneous regions, the corresponding boundary-value problem involves coupled differential equations for the modal expansion coefficients [6]. In surface-wave seismology, the continuous variant has been developed for reference modes, i.e. modes for a reference structure [7] as well as local modes [8,9]. Maupin [10] gives a good review of both alternatives. With a sloping boundary, the continuous variant involves field expansion with modes that do not fulfil the correct boundary conditions. The implied slow convergence of the mode series can be improved by including artificial boundary modes [1,11].

To avoid issues with numerical stability, the two-point boundary-value problem can be recast as an initial-value problem for generalized modal *R*/*T* (reflection/transmission) matrices using invariant embedding. For the continuous coupled-mode variant, this has been done, involving differential equations of Riccati type, for reference modes [7] as well as local modes [12]. There are other ways to achieve numerical stability, e.g. [13], but modal *R*/*T* matrices are crucial in the present paper to separate wavefield components. Hence, the invariant embedding technique is adapted here for discrete coupled local modes using matrix algebra. Explicit computation of transmission matrices is avoided by stabilized back-propagation of modal expansion-coefficient vectors. Mode matching is applied across vertical strip-region boundaries by a mathematically exact Galerkin approach, while, e.g. [14] applies approximate mode matching invoking Snell’s law in each of a number of horizontal sections.

An advantage with the discrete variant of coupled local modes is that the modes need not be tracked carefully as functions of horizontal position to avoid mixing them up. To alleviate this problem for the continuous variant, in the acoustic case with a lossy medium, Pannatoni [15] suggests expansion in terms of local modes for the corresponding lossless medium.

Together with the coupled-mode method, addition rules for *R*/*T* matrices, as developed in [16, Sec. 6.1] and sometimes called Redheffer star products, are used to achieve the desired separation of multiply scattered wavefield components. In essence, known techniques are combined and adapted to treat multiple scattering among laterally displaced medium anomalies in a 2D waveguide.

The application examples connect to recent seismic hazard research concerning amplification of incident plane SH (shear horizontal) waves, and related dynamic stress concentration, caused by 2D medium anomalies close to the surface. Single and multiple inclusions of various types have been considered [17–19], as well as topography irregularities [20] and alluvial valleys [21–23]. Numerical methods like the boundary-element method (BEM), which is used in several of the mentioned papers, are very flexible concerning variations of anomaly shape and type. Ba & Yin [24] present a multidomain BEM for complex local sites, such as a multilayered half-space with inclusions. They decompose the half-space into suitable regions and set up a linear equation system to solve for densities of fictitious uniformly distributed loads on the region boundaries. Shyo & Teng [21] and Kara [22] apply hybrid methods involving finite elements and finite differences, respectively. The spectral element method is another attractive numerical technique that can be applied in this context [25,26].

Special (semi-)analytical methods have been developed for several different kinds of anomalies. There are papers concerning, for example, canyons [27,28], partially filled alluvial valleys [29], hills [30–32] and lined tunnels [33,34]. Media with several anomalies are considered, for example, in [35] (cavities), [36] (canyon and two hills), [37] (hill and cavity), [38] (hill, canyon and tunnel), [39,40] (canyon and cavity/tunnel/inclusion), [41] (alluvial valleys), [42] (hill and canyon), [43] (hills) and [44] (canyon and idealized building). Typically, the wavefield is expanded in terms of appropriate mathematical wave functions in suitable (auxiliary) domains. A region-matching technique allows formulation of the continuity conditions at the domain boundaries as a linear equation system for the expansion coefficients. Point-wise matching (collocation) may be used, or some Galerkin-type method involving transformation and translation formulae between the different wave functions (e.g. Graf’s addition theorem).

An advantage with the coupled-mode approach, compared to the other methods referred to, is the direct applicability of addition rules for *R*/*T* matrices to isolate multiply scattered wavefield components. Compared to the other (semi-)analytical methods mentioned, which are specially designed for particular kinds of anomalies, there is no restriction on the anomaly shapes.

The plan of the paper is as follows. Section 2 introduces wavefield decomposition with partial waves in a medium invariant in one of the horizontal directions. The partial waves isolate various types of multiple scattering among laterally displaced medium anomalies. They are defined using discrete coupled-mode theory and combination of *R*/*T* matrices. This field decomposition is valid for combined P-SV (primary, shear vertical) and SH waves. The remaining text only deals with the pure SH case, however. Section 3 provides a representation of a vertically or obliquely incident plane wave by a horizontal source array. An extension of a Fourier-transform relation involving Bessel functions from Watson [45] appears as a by-product. Section 4 presents details of the discrete coupled-mode method. In essence, this is an adaptation to the 2D SH case of the recent coupled-mode method for 2D fluid media with a three-dimensional (3D) point source in [46, Sec. V]. The adaptation involves, in particular, handling of an incident plane wave by a horizontal source array, and design of the artificial medium termination. Section 5 continues the discussion from §2 of field decomposition into partial waves, allowing isolation of individual (multiply) scattered waves. Reflection-matrix recursion with successive restarts is here an essential ingredient. Section 6 shows how to obtain the full field in a periodic medium efficiently with computations restricted to a single unit cell. A few computational variants are briefly indicated in §7, before some concluding remarks in §8. Two short appendices provide some additions concerning §4.2 for a different type of upper boundary and [46, Sec. V], respectively.

Examples appear in §§4.4, 5.3, 5.4 and 6.2. For convenience, all of them are taken from the recent study of scattering by multiple alluvial valleys in [41]. With the exception of the broad-band example in §5.4, handled by frequency synthesis, a harmonic time dependence according to the typically omitted factor exp(−i*ωt*), where *ω* is the angular frequency and *t* is the time, is assumed throughout the paper.

## 2. Wavefield decomposition in a medium invariant in one of the horizontal directions

**Figure 1.**
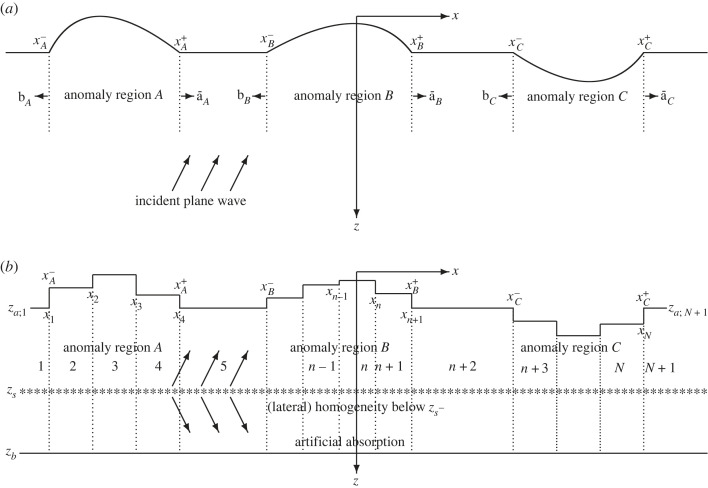
Vertical *xz*-plane with three anomaly regions, *A*, *B* and *C*, in the *y*-independent solid medium. (*a*) An incident plane wave, which is generated by sources at depth *z* = *z*_*s*_ in (*b*). As indicated in (*a*), each anomaly region provides an excitation of its left and right connection regions, producing partial waves for a wavefield decomposition. The anomaly regions are discretized with laterally homogeneous strip regions in (*b*). Strip *n* extends vertically between *z* = *z*_*a*;*n*_ and *z* = *z*_*b*_, *n* = 1, 2, …, *N* + 1. Vertical interfaces at *x* = *x*_1_, *x*_2_, …, *x*_*N*_, indicated by dotted lines, separate the strip regions. The medium anomalies as well as the receivers are above *z*_*s*_−. Below *z*_*s*_−, the medium is typically homogeneous except for the artificial medium truncation.

Consider a solid medium that is invariant in the *y*-direction, where *x*, *y*, *z* are Cartesian coordinates increasing to the east, north and downward, respectively. Below an upper free boundary, the medium agrees, for simplicity, with a certain laterally homogeneous reference structure, except in a number of laterally displaced anomaly regions, denoted *A*, *B*, … and defined by $x_{A}^{-}<x<x_{A}^{+}$, $x_{B}^{-}<x<x_{B}^{+}$, …, respectively. Except in the anomaly regions, the upper boundary is horizontal at a fixed depth. The medium is homogeneous below a certain depth, from where a plane time-harmonic upwards directed wave with direction vector (*k*_*x*_, *k*_*y*_, *k*_*z*_) is incident. Figure 1*a* gives an illustration. The anomalies, i.e. irregularities in the *x*-direction, can involve variations of surface topography or medium parameters (density and velocities) or a combination of both. For ease of illustration, figure 1 shows the first case.

Between two consecutive anomaly regions, possibly also at the left end and at the right end, there is a connection region agreeing, for simplicity, with the reference structure. Denoting the displacement field by **u**(**x**) = (*u*(**x**), *v*(**x**), *w*(**x**))^T^, where **x** = (*x*, *y*, *z*) and *u*, *v*, *w* are the components in the *x*-, *y*-, *z*-directions, respectively, the idea is now to separate different wavefield components or partial waves. A basic partial wave, denoted **u**^0^, is in each anomaly or connection region as if this medium part was embedded within the reference structure. (A laterally homogeneous medium obviously results for a connection region.) Additionally, each anomaly region *A*, *B*, … provides an excitation of its left and right connection regions. In figure 1*a* these excitations, to be more carefully defined in §2.1, are denoted **b**_*X*_ and ${\bar{a}}_{X}$, where *X* = *A*, *B*, … In effect, each anomaly region provides sources for additional partial waves, to be obtained by multiple scattering among the anomaly regions.

Coupled-mode theory provides a useful method to extract these effective sources and additional partial waves. For the discrete variant, consider a discretization of the *y*-independent solid medium by *N* + 1 laterally homogeneous strip regions. With *N* ≥ 0 vertical interfaces *x*_1_ < *x*_2_ < · · · < *x*_*N*_, strip *n* covers *x*_*n*−1_ < *x* < *x*_*n*_ for *n* = 2, 3, …, *N*, while (when *N* > 0) strips 1 and *N* + 1 include *x* < *x*_1_ and *x*_*N*_ < *x*, respectively. (When *N* = 0, strip 1 covers the whole *x*-axis.) The corresponding density, P-wave velocity and shear-wave velocity functions are denoted *ρ*_*n*_(*z*), *α*_*n*_(*z*) and *β*_*n*_(*z*), respectively, for *n* = 1, 2, …, *N* + 1. With absorption in the medium, the velocity functions are complex-valued. Since the upper medium boundary is free, free upper horizontal and vertical boundary segments of the strip regions are also appropriate at the medium discretization, cf. [46, the first two paragraphs of Sec. IV]. The upper horizontal surface of strip *n* is at *z* = *z*_*a*;*n*_. Figure 1*b* provides an illustration, with varying *z* = *z*_*a*;*n*_ to mimic the surface topography in figure 1*a*. The required fineness of the *x*-discretization is related to the wavelength and the incidence directions of the waves [47]. Of course, only the anomaly regions *A*, *B*, … need to be further discretized in this way, each connection region being a single connection strip. For simplicity, the *z*_*a*;*n*_ of the connection strips agree.

To allow a mode representation of the displacement field in each strip region, the medium is artificially truncated with a free horizontal boundary at a finite lower depth *z* = *z*_*b*_. This is the locked-mode medium approximation, used by, e.g. [48,49]. As detailed in §4.1, the medium absorption increases gradually towards the truncation boundary to minimize undesired reflections from it. There is a horizontal plane source array at depth *z* = *z*_*s*_, below all receivers and below all anomalies, to produce the incident plane wave. As indicated in figure 1*b*, the source array also produces a downwards directed plane wave. Below *z*_*s*_ −, the medium is laterally homogeneous. Except for the artificial medium truncation, it is even homogeneous there.

### 2.1. Modal coefficient vectors

In each strip region, the wavefield is expanded in terms of normal modes, with coefficient column vectors **a** or $\bar{a}$ for waves to the right (direction of increasing *x*) and **b** or $\bar{b}$ for waves to the left (direction of decreasing *x*). The coefficient vectors are $\bar{a}$, $\bar{b}$ and **a**, **b** at the left and right ends of the strip, respectively.

In terms of modal coefficient vectors, each anomaly region *X* = *A*, *B*, … provides an excitation of its surroundings with a vector **b**_*X*_ to its connection strip to the left and a vector ${\bar{a}}_{X}$ to its connection strip to the right. These vectors are obtained as the *difference* between the corresponding coefficient vectors computed for the anomaly region embedded within the reference structure and for the reference structure throughout, in both cases ignoring the other anomaly regions. Figure 1*a* gives an illustration.

In effect, these **b**_*X*_ and ${\bar{a}}_{X}$ vectors, one pair for each of the anomaly regions, provide sources for additional partial waves to be computed without further consideration of the sources at *z*_*s*_. Upon propagation through its connection strip, **b**_*X*_ becomes a vector ${\bar{b}}_{X}$ and ${\bar{a}}_{X}$ becomes a vector **a**_*X*_, for excitation of the neighbouring anomaly regions, respectively.

### 2.2. Anomaly regions as two-ports

**Figure 2.**
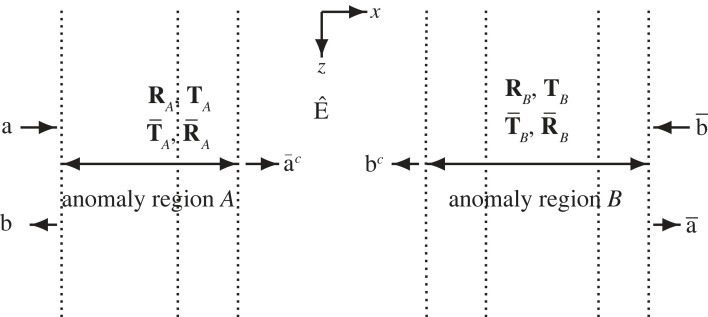
Vertical *xz*-plane with two consecutive anomaly regions *A* and *B* with a connection strip in between. *R*/*T* matrices, input and output field vectors from and to the surrounding connection strips, as well as field vectors for the intermediate connection strip, are indicated.

Disregarding the sources at *z* = *z*_*s*_, consider each anomaly region between two surrounding connection strips as a two-port with input field vectors **a** and $\bar{b}$ from the left and right connection strip, respectively, and corresponding output field vectors **b** and $\bar{a}$ to the left and right connection strip, respectively; cf. figure 2. For the application to a particular partial wave, only one of **a** and $\bar{b}$ is non-vanishing. With appropriate reflection matrices **R**, $\bar{R}$ and transmission matrices **T**, $\bar{T}$, expressing mode conversions,

2.1 $$b=R\cdot a+\bar{T}\cdot \bar{b}\;\mathrm{and}\;\bar{a}=T\cdot a+\bar{R}\cdot \bar{b}.$$

Still disregarding the sources at *z* = *z*_*s*_, consider two consecutive anomaly regions, *A* and *B* for example, with surrounding and intermediate connection strips. The corresponding *R*/*T* matrices are **R**_*A*_, ${\bar{R}}_{A}$, **T**_*A*_, ${\bar{T}}_{A}$ and **R**_*B*_, ${\bar{R}}_{B}$, **T**_*B*_, ${\bar{T}}_{B}$, respectively, and $\hat{E}$ denotes the diagonal transmission matrix of the intermediate connection strip. Figure 2 gives an illustration of this composite structure, with input vectors **a** and $\bar{b}$, and output vectors **b** and $\bar{a}$. These vectors are field vectors in the surrounding connection strips, while ${\bar{a}}^{c}$ and **b**^*c*^ denote field vectors in the intermediate connection strip. Lateral homogeneity of a connection strip allows unambiguous specification of *x*-direction for the waves there. (With corresponding ${\bar{a}}^{c}=0$ or **b**^*c*^ = **0**, a connection strip could be one of the end strips *n* = 1 or *n* = *N* + 1, respectively, of the medium.)

There are well-known addition rules for *R*/*T* matrices [16, Sec. 6.1]. In some texts, they are called Redheffer star products. Concerning **R**, $\bar{R}$, **T**, $\bar{T}$ for the composite structure in figure 2, one obtains

2.2 $$\;\;\begin{array}{rl} R & =R_{A}+{\bar{T}}_{A}\cdot \hat{E}\cdot R_{B}\cdot {\left(I-\hat{E}\cdot {\bar{R}}_{A}\cdot \hat{E}\cdot R_{B}\right)}^{-1}\cdot \hat{E}\cdot T_{A}\\ & =R_{A}+{\bar{T}}_{A}\cdot \hat{E}\cdot R_{B}\cdot \hat{E}\cdot {\left(I-{\bar{R}}_{A}\cdot \hat{E}\cdot R_{B}\cdot \hat{E}\right)}^{-1}\cdot T_{A}, \end{array}$$

2.3 $$\;\begin{array}{rl} T & =T_{B}\cdot {\left(I-\hat{E}\cdot {\bar{R}}_{A}\cdot \hat{E}\cdot R_{B}\right)}^{-1}\cdot \hat{E}\cdot T_{A}\\ & =T_{B}\cdot \hat{E}\cdot {\left(I-{\bar{R}}_{A}\cdot \hat{E}\cdot R_{B}\cdot \hat{E}\right)}^{-1}\cdot T_{A}, \end{array}$$

2.4 $$\begin{array}{rl} \bar{R} & ={\bar{R}}_{B}+T_{B}\cdot \hat{E}\cdot {\bar{R}}_{A}\cdot {\left(I-\hat{E}\cdot R_{B}\cdot \hat{E}\cdot {\bar{R}}_{A}\right)}^{-1}\cdot \hat{E}\cdot {\bar{T}}_{B}\\ & ={\bar{R}}_{B}+T_{B}\cdot \hat{E}\cdot {\bar{R}}_{A}\cdot \hat{E}\cdot {\left(I-R_{B}\cdot \hat{E}\cdot {\bar{R}}_{A}\cdot \hat{E}\right)}^{-1}\cdot {\bar{T}}_{B} \end{array}$$

2.5 $$\begin{array}{rl} \mathrm{and}\;\;\;\;\;\bar{T} & ={\bar{T}}_{A}\cdot {\left(I-\hat{E}\cdot R_{B}\cdot \hat{E}\cdot {\bar{R}}_{A}\right)}^{-1}\cdot \hat{E}\cdot {\bar{T}}_{B}\\ & ={\bar{T}}_{A}\cdot \hat{E}\cdot {\left(I-R_{B}\cdot \hat{E}\cdot {\bar{R}}_{A}\cdot \hat{E}\right)}^{-1}\cdot {\bar{T}}_{B}. \end{array}$$

Concerning the field in the intermediate connection strip, ${\bar{a}}^{c}=T_{A}\cdot a+{\bar{R}}_{A}\cdot \hat{E}\cdot b^{c}$ and $b^{c}={\bar{T}}_{B}\cdot \bar{b}+R_{B}\cdot \hat{E}\cdot {\bar{a}}^{c}$. It follows that

2.6 $${\bar{a}}^{c}={\left(I-{\bar{R}}_{A}\cdot \hat{E}\cdot R_{B}\cdot \hat{E}\right)}^{-1}\cdot (T_{A}\cdot a+{\bar{R}}_{A}\cdot \hat{E}\cdot {\bar{T}}_{B}\cdot \bar{b})$$

and

2.7 $$b^{c}={\left(I-R_{B}\cdot \hat{E}\cdot {\bar{R}}_{A}\cdot \hat{E}\right)}^{-1}\cdot ({\bar{T}}_{B}\cdot \bar{b}+R_{B}\cdot \hat{E}\cdot T_{A}\cdot a).$$

Note the appearance of the same reverberation operators as in the latter alternatives of equations (2.2)–(2.5). Expansion of the reverberation operators, i.e. expansion of the inverse matrices in geometric series [16], provides the basis for the additional partial waves of the field.

With several consecutive anomaly regions, together with surrounding and intermediate laterally homogeneous connection strips, recursive application of equations (2.2)–(2.5) provides *R*/*T* matrices for the composite structure in terms of elementary *R*/*T* matrices for the individual anomaly regions and diagonal transmission matrices for the connection strips. Reflections back and forth between adjacent composite anomaly region structures are readily incorporated. Again, expansion of the reverberation operators provides the basis for the additional partial waves of the field.

### 2.3. Notation for the additional partial waves

As already mentioned, the basic partial wave is denoted **u**^0^. The additional partial waves are denoted $u_{\;j_{l},\ldots ,j_{2},j_{1}}^{\;j_{0}\sigma }$ where σ=− or + and *j*_0_, *j*_1_, …, *j*_*l*_ denote anomaly regions (*A*, *B*, …). A $u_{\;j_{l},\ldots ,j_{2},j_{1}}^{\;j_{0}-}$ wave starts with a $b_{X=j_{0}}$ source vector to the left in the connection strip to the left of anomaly region *j*_0_, while a $u_{\;j_{l},\ldots ,j_{2},j_{1}}^{\;j_{0}+}$ wave starts with an ${\bar{a}}_{X=j_{0}}$ source vector to the right in the connection strip to the right of anomaly region *j*_0_; cf. figure 1*a*. If *l* ≥ 1, the partial wave $u_{\;j_{l},\ldots ,j_{2},j_{1}}^{\;j_{0}\sigma }$ is subsequently reflected when reaching the anomaly regions *j*_1_, *j*_2_, …, *j*_*l*_ in this order, and each of these reflections only involves a single anomaly region. Note that non-adjacent anomaly region indices may here coincide to include all multiple reflections (scattering back and forth).

After its last reflection, the partial wave proceeds by transmission through connection strips and anomaly regions towards the left or right end of the medium, thereby contributing to the field. In a connection strip, only waves to the left or right contribute; waves in the opposite direction appear in other partial waves. Within an anomaly region, however, the transmission gives rise to interior reflections which contribute as well.

The total field in a particular anomaly region or connection strip appears by summing the contributions there by **u**^0^ and the additional partial waves. There is no high-frequency ray approximation, and the field decomposition is mathematically exact.

### 2.4. The pure SH case

So far, the discussion of the waves in the *y*-independent solid medium has been rather general. For simplicity, however, the following development is restricted to the pure SH wave case without conversions between SH (Love) and P-SV (Rayleigh) modes at the vertical *x* = *x*_*n*_ interfaces. Specifically, *k*_*y*_ = 0 for the incident plane wave and **u**(**x**) = **u**(*x*,*z*) = (0, *v*(*x*,*z*), 0)^T^. Denoting the medium density and shear-wave velocity by *ρ*(*x*,*z*) and *β*(*x*,*z*), respectively, and introducing the corresponding Lamé parameter *μ*(*x*,*z*) = *ρ*(*x*,*z*) *β*^2^(*x*,*z*), the *y*-component *v*(*x*,*z*) of the displacement satisfies the SH-wave Helmholtz equation:

2.8 $$\Delta v+\mu^{-1}\;\mathrm{grad}\;\mu \cdot \mathrm{grad}\;v+{\left(\frac{\omega }{\beta }\right)}^{2}v=-\mu^{-1}f.$$

Here, *f*(*x*,*z*) is the *y*-component of the body force per unit volume, Δ is the Laplacian, and grad is the gradient operator. Moreover, the components *τ*_*xy*_ and *τ*_*yz*_ of the stress tensor *τ* appear as *τ*_*xy*_ = *μ* ∂*v*/∂*x* and *τ*_*yz*_ = *μ* ∂*v*/∂*z*. This follows readily from basic linear elasticity theory (e.g. [50, ch. 2]). The medium velocity *β* may be complex-valued to accommodate absorption.

The coupled-mode SH-wave method of the present paper is related to the method for the fluid case in [46, Sec. V]. Actually, a fluid-medium analogy of the SH case appears by setting the density to 1/*μ* (possibly complex-valued), the sound speed to *β*, and the pressure to *v*. A free/rigid solid-medium boundary (with vanishing body force) with normal in the *xz*-plane then corresponds to a rigid/free fluid-medium boundary.

## 3. Generating a plane SH wave by a line array of line sources

The development in [46, Sec. V] concerns a 3D point source (or line source), while SH papers such as [27,41] typically prescribe an incident plane wave. Generation of a plane wave by an array of point or line sources in a homogeneous solid medium with shear-wave velocity *β* is now considered.

At first, consider equation (2.8) with its right-hand side replaced by $r_{\mathrm{ref}}^{2}\delta (x-x_{s})\delta (y-y_{s})\delta (z-z_{s})$ for a 3D point source at **x**_*s*_ = (*x*_*s*_, *y*_*s*_, *z*_*s*_). Here, *δ* is the one-dimensional Dirac delta function with dimension m^−1^, and *r*_ref_ is a reference length, introduced to achieve consistency of dimensions. The solution for *v*(*x*,*y*,*z*), now depending on *y* as well because of the 3D point source, becomes (e.g. [51, Sec. 4-3])

3.1 $$v(x)=-r_{\mathrm{ref}}^{2}\frac{\exp(i|x-x_{s}|\omega /\beta )}{4\pi |x-x_{s}|}.\;$$

Next, consider a horizontal line source in the *y*-coordinate direction, according to *r*_ref_ *δ*(*x* − *x*_*s*_) *δ*(*z* − *z*_*s*_) in the right-hand side of equation (2.8). The solution for the displacement *v*(*x*,*z*) in the *y*-direction becomes

3.2 $$v(x,z)=-\frac{i}{4}r_{\mathrm{ref}}H_{0}^{\left(1\right)}\left(\frac{\omega r}{\beta }\right),$$

where $r=\sqrt{{\left(x-x_{s}\right)}^{2}+{\left(z-z_{s}\right)}^{2}}$ (e.g. [4, Sec. 5.2.2]). A verification can be obtained by integrating point-source solutions according to equation (3.1) over *y*_*s*_ and applying an integral representation of the Hankel function [52, 9.1.24].

The solution for a horizontal plane source according to exp(i*k*_*x*_*x*) *δ*(*z* − *z*_*s*_) in the right-hand side of equation (2.8) is

3.3 $$v(x,z)=-\frac{i}{2}\frac{\exp\left[i(k_{x}x+|z-z_{s}|\sqrt{\omega^{2}/\beta^{2}-k_{x}^{2}})\right]}{\sqrt{\omega^{2}/\beta^{2}-k_{x}^{2}}},$$

where the square root is taken with a non-negative imaginary part. This is readily seen by isolating the dependence on *x* according to the factor exp(i*k*_*x*_*x*), applying the radiation conditions and observing the implied step discontinuity of ∂*v*/∂*z* across *z* = *z*_*s*_. Originating from *z* = *z*_*s*_, cf. figure 1*b*, there is obviously an upwards directed plane wave and a downwards directed one.

The solution according to equation (3.3) can be obtained in two other ways: by integration of line sources over a line or point sources over a plane. Concerning the first alternative, an application of

3.4 $$e^{ik_{x}x}\delta (z-z_{s})=\int_{-\infty }^{+\infty }\;e^{ik_{x}x_{s}}\delta (x-x_{s})\delta (z-z_{s})\;dx_{s}$$

and equation (3.2) yields

3.5 $$v(x,z)=-\frac{i}{4}\int_{-\infty }^{+\infty }H_{0}^{\left(1\right)}\left(\frac{\omega }{\beta }\sqrt{{\left(x-x_{s}\right)}^{2}+{\left(z-z_{s}\right)}^{2}}\right)\;e^{ik_{x}x_{s}}\;dx_{s}.$$

Together with equation (3.3), and the inverse Fourier transform, equation (3.5) verifies an instance of Bostrőm *et al.* [53, eqn (4.13)].

Concerning the second alternative, an application of

3.6 $$e^{ik_{x}x}\delta (z-z_{s})=\int_{-\infty }^{+\infty }\;e^{ik_{x}x_{s}}\;dx_{s}\int_{-\infty }^{+\infty }\delta (x-x_{s})\delta (y-y_{s})\delta (z-z_{s})\;dy_{s}$$

and equation (3.1) together with a polar transformation of variables (*x*_*s*_ = *x* + *r* cos*θ*, *y*_*s*_ = *y* + *r* sin*θ*) yields

3.7 $$v(x,z)=-\frac{1}{2}e^{ik_{x}x}\int_{0}^{+\infty }J_{0}(k_{x}r)\frac{\exp[i(\omega /\beta )\sqrt{r^{2}+{\left(z-z_{s}\right)}^{2}}]}{\sqrt{r^{2}+{\left(z-z_{s}\right)}^{2}}}r\;dr.$$

**Proposition 3.1.**

With *k* = *ω*/*β*, equations (3.3) and (3.7) apparently show that

3.8 $$\begin{array}{rl} \int_{0}^{+\infty }J_{0}(k_{x}r)\frac{\exp\left[ik\sqrt{r^{2}+z^{2}}\right]}{\sqrt{r^{2}+z^{2}}}r\;dr & =\int_{\left|z\right|}^{+\infty }J_{0}\left(k_{x}\sqrt{t^{2}-z^{2}}\right)\;e^{ikt}\;dt\\ & =i\frac{\exp\left[i(|z|\sqrt{k^{2}-k_{x}^{2}})\right]}{\sqrt{k^{2}-k_{x}^{2}}}. \end{array}$$

By analytic continuation, this relation is valid for complex *k* and *k*_*x*_ such that |Im(*k*_*x*_)| < Im(*k*). It is also valid when |Im(*k*_*x*_)| = Im(*k*), provided that *k*_*x*_ ≠ ±*k* and that Re(*k*) > 0 if Im(*k*) = 0. As before, the square roots are taken with non-negative imaginary parts.

Equation (3.8) represents an extension, to non-vanishing *z*, of Watson [45, eqn (1) in §13.2].

## 4. Coupled-mode SH-wave computations

Consider figure 1*b* with a discretization of the *y*-independent solid medium into *N* + 1 laterally homogeneous strip regions. Let $\mu_{n}(z)=\rho_{n}(z)\beta_{n}^{2}(z)$, *n* = 1, 2, …, *N* + 1. Before returning to the partial waves in §5, the present §4 deals with coupled-mode computation of the full wavefield.

There is a horizontal plane source array at *z* = *z*_*s*_ corresponding to the right-hand side

4.1 $$\varphi (x)\delta (z-z_{s})=\int_{-\infty }^{+\infty }\varphi (x_{s})\delta (x-x_{s})\delta (z-z_{s})\;dx_{s}$$

in equation (2.8). This is a slight generalization of the particular choice φ(*x*) = exp(i*k*_*x*_*x*) in §3. As in §2, the depth *z*_*s*_ is below all receivers and below all anomalies (irregularities in the *x*-direction), such that the medium is laterally homogeneous below *z*_*s*_ −.

For *n* = 1, 2, …, *N* + 1, let *k*_*m*,*n*_ and *Z*_*m*,*n*_(*z*), *m* = 1, 2, …, denote the modal horizontal wavenumbers and normalized mode functions, respectively, for SH modes in strip region *n*. The wavenumbers *k*_*m*,*n*_ appear in the upper complex plane but not on the negative real axis. It is appropriate to order them according to decreasing real parts and (close to the imaginary axis) increasing imaginary parts. The mode normalization means that *Z*_*m*,*n*_(*z*) denotes the original mode function $Z_{m,n}^{0}(z)$ divided by ${\left[\int_{z_{a;n}}^{z_{b}}\mu_{n}(\zeta )(Z_{m,n}^{0}{\left(\zeta )\right)}^{2}\;d\zeta \right]}^{1/2}$. According to Sturm–Liouville theory, the modes in strip *n* are orthogonal with respect to the weight function *μ*_*n*_(*z*). Well established methods exist to compute the *k*_*m*,*n*_ and the *Z*_*m*,*n*_(*z*) (e.g. [4,54]). For robust and automatic computations of the *k*_*m*,*n*_ with winding-number integrals, it can be convenient to use quadruple precision.

Introduce row vectors $\Phi_{n}{\left(x,z)=\{(\Phi_{n}(x,z)\right)}_{m}\}$ and $\Psi_{n}(x,z)=\{{\left(\Psi_{n}(x,z)\right)}_{m}\}$ for basic solutions of the homogeneous version of equation (2.8) in the different strips *n* = 1, 2, …, *N* + 1. Specifically,

4.2 $$\left(\Phi_{n}{\left(x,z)\right)}_{m}=\frac{1}{k_{m,n}}Z_{m,n}(z){\hat{E}}_{m,n}^{\left(1\right)}(x\right)$$

and

4.3 $${\left(\Psi_{n}(x,z)\right)}_{m}=Z_{m,n}(z){\hat{E}}_{m,n}^{\left(2\right)}(x),$$

where

4.4 $${\hat{E}}_{m,n}^{\left(1\right)}(x)=\exp(ik_{m,n}(x-x_{n-1}))\;\mathrm{with}\;{\hat{E}}_{m,n}^{\left(1\right)}(x_{n-1})=1$$

and

4.5 $${\hat{E}}_{m,n}^{\left(2\right)}(x)=\exp(-ik_{m,n}(x-x_{n}))\;\mathrm{with}\;{\hat{E}}_{m,n}^{\left(2\right)}(x_{n})=1,$$

*x*_0_ = *x*_1_, and *x*_*N*+1_ = *x*_*N*_. (When *N* = 0, *x*_1_ = 0 m.) The factor 1/*k*_*m*,*n*_ in the definition of ${\left(\Phi_{n}(x,z)\right)}_{m}$ is a kind of normalization, cf. the normalization of the modes. It leads to different dimensions of $\Phi_{n}(x,z)$ and $\Psi_{n}(x,z)$, but it simplifies some forthcoming equations and it makes the appearing reflection matrices (see §4.2) symmetric. Additionally, introduce the diagonal matrices ${\hat{E}}_{n}={\mathrm{diag}}_{m}({\hat{E}}_{m,n}^{\left(1\right)}(x_{n}))=$ ${\mathrm{diag}}_{m}({\hat{E}}_{m,n}^{\left(2\right)}(x_{n-1}))$ for transmission, or transfer of *x*-reference, between the sides of strip region *n*. Note that ${\hat{E}}_{1}={\hat{E}}_{N+1}=I$.

At each *x*, expand the field in terms of the local modes there. It follows, cf. [46, eqns (35)–(36)], that there are coefficient column vectors ${\bar{a}}_{n}=\{{\left({\bar{a}}_{n}\right)}_{m}\}$, ${\bar{a}}_{n}^{L}=\{{\left({\bar{a}}_{n}^{L}\right)}_{m}\}$, ${\bar{a}}_{n}^{R}=\{{\left({\bar{a}}_{n}^{R}\right)}_{m}\}$ for waves to the right (increasing *x*) with *x*-reference at *x*_*n*−1_, and **b**_*n*_ = {(*b*_*n*_)_*m*_}, $b_{n}^{L}=\{{\left(b_{n}^{L}\right)}_{m}\}$, $b_{n}^{R}=\{{\left(b_{n}^{R}\right)}_{m}\}$ for waves to the left (decreasing *x*) with *x*-reference at *x*_*n*_, such that, within strip region *n*,

4.6 $$\begin{array}{rl} v(x,z) & =\Phi_{n}(x,z)\cdot ({\bar{a}}_{n}+{\bar{a}}_{n}^{L}+{\bar{a}}_{n}^{R})+\Psi_{n}(x,z)\cdot (b_{n}+b_{n}^{L}+b_{n}^{R})\\ & \;+\sum_{m}a_{m,s}Z_{m,n}(z)\int_{J_{n}}\varphi (x_{s})\frac{\exp(ik_{m,n}|x-x_{s}|)}{k_{m,n}}\;dx_{s}, \end{array}$$

where *J*_*n*_ = (*x*_*n*−1_, *x*_*n*_) for *n* = 2, 3, …, *N*, *J*_1_ = (−∞, *x*_1_) and *J*_*N*+1_ = (*x*_*N*_, +∞) when *N* > 0, *J*_1_ = (−∞, +∞) when *N* = 0, and

4.7 $$a_{m,s}=-\frac{i}{2}\mu_{n}(z_{s})Z_{m,n}(z_{s}).$$

In equation (4.6), ${\bar{a}}_{n}^{L}$ and $b_{n}^{L}$ represent contributions from sources to the left of strip *n*, while ${\bar{a}}_{n}^{R}$ and $b_{n}^{R}$ represent contributions from sources to the right of strip *n*. Hence, ${\bar{a}}_{1}^{L}=b_{1}^{L}={\bar{a}}_{N+1}^{R}=b_{N+1}^{R}=0$. Moreover, ${\bar{a}}_{1}={\bar{a}}_{1}^{R}=b_{N+1}=b_{N+1}^{L}=0$ are boundary conditions. The mode sum in equation (4.6) follows by equation (4.1) and integration over *x*_*s*_ of basic modal line-source solutions according to, e.g. [4, Sec. 5.2.2].

Section 4.1 gives some details on the medium truncation. Subsequently, essentially generalizing the development in [46, Sec. V] to a continuous source array, §§4.2 and 4.3 present a coupled-mode method to determine the column vectors ${\bar{a}}_{n}$, ${\bar{a}}_{n}^{L}$, ${\bar{a}}_{n}^{R}$ and **b**_*n*_, $b_{n}^{L}$, $b_{n}^{R}$. Of course, only a finite number of modes is kept in each strip at the actual computations.

### 4.1. Artificial medium truncation

The issue here, cf. [46, Sec. IV E], is to design an artificial medium truncation, ending at *z* = *z*_*b*_, providing negligible reflections. Recalling figure 1*b* and equation (3.3), the reflections from the downwards directed plane wave must be minimized. Moreover, there are undesired reflections at depth from the downwards directed surface reflections of the upwards directed plane wave. Because of the medium anomalies, these waves may have a slightly different incidence angle onto the artificial medium truncation. Finally, waves are of course scattered downwards in various directions from the anomalies.

The most important incidence angle, or *k*_*x*_, to be handled by the artificial medium truncation is obviously that of the downwards directed plane wave. For rapid truncation-design computations, consider two related variants of the medium, with source array according to exp(i*k*_*x*_*x*) *δ*(*z* − *z*_*s*_) in the right-hand side of equation (2.8): (i) a homogeneous medium with density *ρ*^0^, shear-wave velocity *β*^0^ and *v*(*x*,*z*) given by equation (3.3) with *β* = *β*^0^, and (ii) its modification with laterally homogeneous artificial medium truncation including absorption between *z* = *z*_*s*_ and *z* = *z*_*b*_.

For medium (ii), *β* (and *μ*) depend on *z* below *z* = *z*_*s*_. It follows that *v*(*x*,*z*) = exp(i*k*_*x*_*x*) *Z*(*z*), where *Z*(*z*) fulfils

4.8 $$Z(z)=-\gamma \frac{i}{2}\frac{\exp(i|z-z_{s}|\sqrt{\omega^{2}/{\left(\beta^{0}\right)}^{2}-k_{x}^{2}})}{\sqrt{\omega^{2}/{\left(\beta^{0}\right)}^{2}-k_{x}^{2}}}$$

and

4.9 $$Z^{\prime \prime }(z)+\frac{\mu^{\prime }(z)}{\mu (z)}Z^{\prime }(z)+\left(\frac{\omega^{2}}{\beta^{2}(z)}-k_{x}^{2}\right)Z(z)=0$$

for *z* < *z*_*s*_ and *z*_*s*_ < *z* < *z*_*b*_, respectively. Here, *μ*(*z*) = *ρ*^0^ *β*^2^(*z*). In addition, allowing numerical determination of the constant *γ* for specified complex-valued *β*(*z*) and a real *k*_*x*_, there is the source-discontinuity condition *Z*′(*z*_*s*+_) − *Z*′(*z*_*s*−_) = 1 and the free-boundary condition *Z*′(*z*_*b*_) = 0.

For an appropriate medium truncation, *γ* should apparently be close to 1, making the solutions for media (i) and (ii) similar for *z* < *z*_*s*_. For a trial *z*_*b*_, evolutionary optimization algorithms, such as differential evolution, are useful to minimize |*γ* − 1| by varying *β*(*z*). It is thereby convenient to specify the complex-valued function *β*(*z*) by the following three design parameters: the depth between *z*_*s*_ and *z*_*b*_ for onset of artificial absorption, *β*(*z*_*b*_), and the polynomial degree of the change towards *z*_*b*_ (1 for linear change) of *β*^−2^(*z*). Of course, a large *z*_*b*_ allows small artificial reflections, but it necessitates a large number of normal modes for the field representation. Hence, *z*_*b*_ (as well as *z*_*s*_) should be reasonably small, while still providing a negligible |*γ* − 1| at the minimization.

Numerical experiments with different *z*_*b*_ are useful. Small incidence angles (close to normal incidence) may allow a small *z*_*b*_ with a large absorption gradient from a shallow onset, while large incidence angles (close to grazing incidence) may require a large *z*_*b*_ with a smooth absorption increase. Recalling that waves from the anomalies may be scattered in various directions onto the artificial medium truncation, it is a good idea to include several incidence angles, i.e. several *k*_*x*_ and *γ*, in the minimization.

### 4.2. Reflection-matrix recursion

By physical arguments, considering $\Phi_{n}$ waves to the right and $\Psi_{n}$ waves to the left, there must exist modal reflection matrices **R**_*n*_ with *x*-reference at *x*_*n*_ and ${\bar{R}}_{n}$ with *x*-reference at *x*_*n*−1_, which are independent of the sources, such that, for *n* = 1, 2, …, *N* + 1,

4.10 $$b_{n}^{L}=R_{n}\cdot {\hat{E}}_{n}\cdot {\bar{a}}_{n}^{L}\;\mathrm{and}\;b_{n}=R_{n}\cdot a_{n}$$

and

4.11 $${\bar{a}}_{n}^{R}={\bar{R}}_{n}\cdot {\hat{E}}_{n}\cdot b_{n}^{R}\;\mathrm{and}\;{\bar{a}}_{n}={\bar{R}}_{n}\cdot {\bar{b}}_{n}.$$

Accounting for changes of *x*-reference from *x*_*n*−1_ to *x*_*n*_ and vice versa, cf. [46, eqns (37)–(38)],

4.12 $$a_{n}={\hat{E}}_{n}\cdot {\bar{a}}_{n}-\frac{i}{2}\mu_{n}(z_{s})\int_{J_{n}}\varphi (x_{s})\Psi_{n}^{T}(x_{s},z_{s})\;dx_{s}$$

and

4.13 $${\bar{b}}_{n}={\hat{E}}_{n}\cdot b_{n}-\frac{i}{2}\mu_{n}(z_{s})\int_{J_{n}}\varphi (x_{s})\Phi_{n}^{T}(x_{s},z_{s})\;dx_{s},$$

respectively. Note that equations (4.12) and (4.13) are irrelevant when *n* = *N* + 1 and *n* = 1, respectively, since $R_{N+1}={\bar{R}}_{1}=0$. With the chosen normalization of the normal modes and the basic wave functions, reciprocity arguments of the same type as in [46, Sec. VI] show that all modal reflection matrices **R**_*n*_ and ${\bar{R}}_{n}$ are symmetric.

The integrals in equations (4.6) and (4.12)–(4.13) are easy to compute analytically when φ(*x*) = exp(i*k*_*x*_*x*). Note that the wavenumbers *k*_*m*,*n*_ have positive imaginary parts because of the artificial absorption above *z*_*b*_.

Corresponding to the Riccati-equation solutions for the related continuous coupled-mode approach in [7], the modal reflection matrices **R**_*n*_ and ${\bar{R}}_{n}$ may be computed recursively, for decreasing *n* starting with **R**_*N*+1_ = **0** and for increasing *n* starting with ${\bar{R}}_{1}=0$, respectively. The recursion equations are derived, by a Galerkin approach, from the continuity of *v* and *τ*_*xy*_ at the vertical interfaces separating the strip regions, cf. [55, Secs. III A,D; 46, Secs. IV A-D]. Specifically, let *I*_*n*_ denote the depth interval [*z*_*a*;*n*_, *z*_*b*_]. Introduce the mode-coupling matrices **F**_*n*_, **G**_*n*_ and ${\bar{F}}_{n}$, ${\bar{G}}_{n}$ with elements (*m*, *m*′) given by

4.14 $${\left(F_{n}\right)}_{m,m^{\prime }}=\int_{I_{n+1}\cap I_{n}}\mu_{n+1}(z)Z_{m,n+1}(z)Z_{m^{\prime },n}(z)\;dz,$$

4.15 $${\left(G_{n}\right)}_{m,m^{\prime }}=\frac{k_{m^{\prime },n}}{k_{m,n+1}}\int_{I_{n+1}\cap I_{n}}\mu_{n}(z)Z_{m,n+1}(z)Z_{m^{\prime },n}(z)\;dz,$$

4.16 $${\left({\bar{F}}_{n}\right)}_{m,m^{\prime }}=\int_{I_{n-1}\cap I_{n}}\mu_{n-1}(z)Z_{m,n-1}(z)Z_{m^{\prime },n}(z)\;dz$$

4.17 $$\mathrm{and}\;{\left({\bar{G}}_{n}\right)}_{m,m^{\prime }}=\frac{k_{m^{\prime },n}}{k_{m,n-1}}\int_{I_{n-1}\cap I_{n}}\mu_{n}(z)Z_{m,n-1}(z)Z_{m^{\prime },n}(z)\;dz.$$

Note that $I_{n\pm 1}\cap I_{n}=[\max(z_{a;n\pm 1},z_{a;n}),z_{b}]$. In addition, define the diagonal matrices **K**_*n*_ by **K**_*n*_ = diag_*m*_ (*k*_*m*,*n*_).

#### 4.2.1. Recursion of **R**_*n*_ for decreasing *n*

By the boundary conditions, *τ*_*xy*_ = *μ* ∂*v*/∂*x* vanishes when *x* = *x*_*n*_ and min(*z*_*a*;*n*_, *z*_*a*;*n*+1_) < *z* < max(*z*_*a*;*n*_, *z*_*a*;*n*+1_). Introduce the notation

4.18 $$a_{n}^{L+}=a_{n}+{\hat{E}}_{n}\cdot {\bar{a}}_{n}^{L}\;\mathrm{and}\;b_{n}^{L+}=b_{n}+b_{n}^{L},$$

and disregard temporarily sources to the right of *x* = *x*_*n*_.

When $I_{n+1}⊇I_{n}$, equation (4.6) provides, together with depth integrations over *I*_*n*_ and *I*_*n*+1_,

4.19 $${\bar{F}}_{n+1}\cdot (K_{n+1}^{-1}\cdot {\bar{a}}_{n+1}^{L}+{\hat{E}}_{n+1}\cdot b_{n+1}^{L})=K_{n}^{-1}\cdot a_{n}^{L+}+b_{n}^{L+}$$

and

4.20 $$K_{n+1}^{-1}\cdot {\bar{a}}_{n+1}^{L}-{\hat{E}}_{n+1}\cdot b_{n+1}^{L}=G_{n}\cdot (K_{n}^{-1}\cdot a_{n}^{L+}-b_{n}^{L+})$$

for continuity at *x*_*n*_ of *v* after multiplication with *μ*_*n*_(*z*)*Z*_*m*,*n*_(*z*) and *τ*_*xy*_ after multiplication with *Z*_*m*,*n*+1_(*z*), respectively. Insertion of the relation $b_{n+1}^{L}=R_{n+1}\cdot {\hat{E}}_{n+1}\cdot {\bar{a}}_{n+1}^{L}$ from equation (4.10) yields, after elimination of $b_{n}^{L+}$,

4.21 $$K_{n+1}^{-1}\cdot {\bar{a}}_{n+1}^{L}=2W_{n}^{-1}\cdot G_{n}\cdot K_{n}^{-1}\cdot a_{n}^{L+}\;\mathrm{where}$$

4.22 $$W_{n}=I+G_{n}\cdot {\bar{F}}_{n+1}-(I-G_{n}\cdot {\bar{F}}_{n+1})\cdot {\hat{E}}_{n+1}\cdot R_{n+1}\cdot K_{n+1}\cdot {\hat{E}}_{n+1}.$$

Together with $b_{n}^{L+}=R_{n}\cdot a_{n}^{L+}$, equation (4.19) subsequently provides the reflection-matrix recursion equation:

4.23 $$R_{n}\cdot K_{n}=-I+2{\bar{F}}_{n+1}\cdot (I+{\hat{E}}_{n+1}\cdot R_{n+1}\cdot K_{n+1}\cdot {\hat{E}}_{n+1})\cdot W_{n}^{-1}\cdot G_{n}.$$

When *I*_*n*+1_⊆ *I*_*n*_, on the other hand, integrations over *I*_*n*+1_ and *I*_*n*_ provide

4.24 $$K_{n+1}^{-1}\cdot {\bar{a}}_{n+1}^{L}+{\hat{E}}_{n+1}\cdot b_{n+1}^{L}=F_{n}\cdot (K_{n}^{-1}\cdot a_{n}^{L+}+b_{n}^{L+})$$

and

4.25 $${\bar{G}}_{n+1}\cdot (K_{n+1}^{-1}\cdot {\bar{a}}_{n+1}^{L}-{\hat{E}}_{n+1}\cdot b_{n+1}^{L})=K_{n}^{-1}\cdot a_{n}^{L+}-b_{n}^{L+}$$

for continuity of *v* after multiplication with *μ*_*n*+1_(*z*)*Z*_*m*,*n*+1_(*z*) and *τ*_*xy*_ after multiplication with *Z*_*m*,*n*_(*z*), respectively. This yields

4.26 $$K_{n+1}^{-1}\cdot {\bar{a}}_{n+1}^{L}=2W_{n}^{-1}\cdot F_{n}\cdot K_{n}^{-1}\cdot a_{n}^{L+}\;\mathrm{where}\;\;\mathrm{now}$$

4.27 $$W_{n}=I+F_{n}\cdot {\bar{G}}_{n+1}+(I-F_{n}\cdot {\bar{G}}_{n+1})\cdot {\hat{E}}_{n+1}\cdot R_{n+1}\cdot K_{n+1}\cdot {\hat{E}}_{n+1}.$$

The equation for reflection-matrix recursion follows from equation (4.25) as

4.28 $$R_{n}\cdot K_{n}=I-2{\bar{G}}_{n+1}\cdot (I-{\hat{E}}_{n+1}\cdot R_{n+1}\cdot K_{n+1}\cdot {\hat{E}}_{n+1})\cdot W_{n}^{-1}\cdot F_{n}.$$

When *I*_*n*+1_ = *I*_*n*_, a third option is possible by combining equations (4.24) and (4.20). This leads to, cf. [55, Sec. III A],

4.29 $$K_{n+1}^{-1}\cdot {\bar{a}}_{n+1}^{L}=\frac{1}{2}[F_{n}+G_{n}+(F_{n}-G_{n})\cdot R_{n}\cdot K_{n}]\cdot K_{n}^{-1}\cdot a_{n}^{L+}$$

and

4.30 $$\begin{array}{rl} R_{n}\cdot K_{n} & =-{\left[F_{n}+G_{n}-{\hat{E}}_{n+1}\cdot R_{n+1}\cdot K_{n+1}\cdot {\hat{E}}_{n+1}\cdot (F_{n}-G_{n})\right]}^{-1}\\ & \;\cdot [F_{n}-G_{n}-{\hat{E}}_{n+1}\cdot R_{n+1}\cdot K_{n+1}\cdot {\hat{E}}_{n+1}\cdot (F_{n}+G_{n})]. \end{array}$$

This third option is more efficient computationally, but it requires that the kept finite numbers of modes in the two adjacent strips are equal.

#### 4.2.2. Recursion of ${\bar{R}}_{n}$ for increasing *n*

By the boundary conditions, *τ*_*xy*_ vanishes when *x* = *x*_*n*−1_ and min(*z*_*a*;*n*_, *z*_*a*;*n*−1_) < *z* < max(*z*_*a*;*n*_, *z*_*a*;*n*−1_). Introduce the notation

4.31 $${\bar{b}}_{n}^{R+}={\bar{b}}_{n}+{\hat{E}}_{n}\cdot b_{n}^{R}\;\mathrm{and}\;{\bar{a}}_{n}^{R+}={\bar{a}}_{n}+{\bar{a}}_{n}^{R},$$

and disregard now temporarily sources to the left of *x* = *x*_*n*−1_.

When $I_{n-1}⊇I_{n}$, equation (4.6) provides, together with depth integrations over *I*_*n*_ and *I*_*n*−1_,

4.32 $$F_{n-1}\cdot (b_{n-1}^{R}+{\hat{E}}_{n-1}\cdot K_{n-1}^{-1}\cdot {\bar{a}}_{n-1}^{R})={\bar{b}}_{n}^{R+}+K_{n}^{-1}\cdot {\bar{a}}_{n}^{R+}$$

and

4.33 $$b_{n-1}^{R}-{\hat{E}}_{n-1}\cdot K_{n-1}^{-1}\cdot {\bar{a}}_{n-1}^{R}={\bar{G}}_{n}\cdot ({\bar{b}}_{n}^{R+}-K_{n}^{-1}\cdot {\bar{a}}_{n}^{R+})$$

for continuity at *x*_*n*−1_ of *v* after multiplication with *μ*_*n*_(*z*)*Z*_*m*,*n*_(*z*) and *τ*_*xy*_ after multiplication with *Z*_*m*,*n*−1_(*z*), respectively. Insertion of the relation ${\bar{a}}_{n-1}^{R}={\bar{R}}_{n-1}\cdot {\hat{E}}_{n-1}\cdot b_{n-1}^{R}$ from equation (4.11) yields, after elimination of $K_{n}^{-1}\cdot {\bar{a}}_{n}^{R+}$,

4.34 $$b_{n-1}^{R}=2\;W_{n}^{-1}\cdot {\bar{G}}_{n}\cdot {\bar{b}}_{n}^{R+}\;\mathrm{where}\;\;\mathrm{now}$$

4.35 $$W_{n}=I+{\bar{G}}_{n}\cdot F_{n-1}-(I-{\bar{G}}_{n}\cdot F_{n-1})\cdot {\hat{E}}_{n-1}\cdot K_{n-1}^{-1}\cdot {\bar{R}}_{n-1}\cdot {\hat{E}}_{n-1}.$$

Since ${\bar{a}}_{n}^{R+}={\bar{R}}_{n}\cdot {\bar{b}}_{n}^{R+}$, the equation for reflection-matrix recursion follows as

4.36 $$K_{n}^{-1}\cdot {\bar{R}}_{n}=-I+2\;F_{n-1}\cdot (I+{\hat{E}}_{n-1}\cdot K_{n-1}^{-1}\cdot {\bar{R}}_{n-1}\cdot {\hat{E}}_{n-1})\cdot W_{n}^{-1}\cdot {\bar{G}}_{n}.$$

When *I*_*n*−1_⊆ *I*_*n*_, on the other hand, integrations over *I*_*n*−1_ and *I*_*n*_ provide

4.37 $$b_{n-1}^{R}+{\hat{E}}_{n-1}\cdot K_{n-1}^{-1}\cdot {\bar{a}}_{n-1}^{R}={\bar{F}}_{n}\cdot ({\bar{b}}_{n}^{R+}+K_{n}^{-1}\cdot {\bar{a}}_{n}^{R+})$$

and

4.38 $$G_{n-1}\cdot (b_{n-1}^{R}-{\hat{E}}_{n-1}\cdot K_{n-1}^{-1}\cdot {\bar{a}}_{n-1}^{R})={\bar{b}}_{n}^{R+}-K_{n}^{-1}\cdot {\bar{a}}_{n}^{R+}$$

for continuity of *v* after multiplication with *μ*_*n*−1_(*z*)*Z*_*m*,*n*−1_(*z*) and *τ*_*xy*_ after multiplication with *Z*_*m*,*n*_(*z*), respectively. This yields

4.39 $$b_{n-1}^{R}=2W_{n}^{-1}\cdot {\bar{F}}_{n}\cdot {\bar{b}}_{n}^{R+}\;\mathrm{where}\;\;\mathrm{now}$$

4.40 $$W_{n}=I+{\bar{F}}_{n}\cdot G_{n-1}+(I-{\bar{F}}_{n}\cdot G_{n-1})\cdot {\hat{E}}_{n-1}\cdot K_{n-1}^{-1}\cdot {\bar{R}}_{n-1}\cdot {\hat{E}}_{n-1}.$$

The equation for reflection-matrix recursion becomes

4.41 $$K_{n}^{-1}\cdot {\bar{R}}_{n}=I-2G_{n-1}\cdot (I-{\hat{E}}_{n-1}\cdot K_{n-1}^{-1}\cdot {\bar{R}}_{n-1}\cdot {\hat{E}}_{n-1})\cdot W_{n}^{-1}\cdot {\bar{F}}_{n}.$$

When *I*_*n*−1_ = *I*_*n*_, and the numbers of modes kept in the two strips *n* − 1 and *n* are equal, a third option is possible by combining equations (4.37) and (4.33). This leads to, cf. [55, Sec. III D],

4.42 $$b_{n-1}^{R}=\frac{1}{2}[{\bar{F}}_{n}+{\bar{G}}_{n}+({\bar{F}}_{n}-{\bar{G}}_{n})\cdot K_{n}^{-1}\cdot {\bar{R}}_{n}]\cdot {\bar{b}}_{n}^{R+}$$

and

4.43 $$\begin{array}{rl} K_{n}^{-1}\cdot {\bar{R}}_{n} & =-{\left[{\bar{F}}_{n}+{\bar{G}}_{n}-{\hat{E}}_{n-1}\cdot K_{n-1}^{-1}\cdot {\bar{R}}_{n-1}\cdot {\hat{E}}_{n-1}\cdot ({\bar{F}}_{n}-{\bar{G}}_{n})\right]}^{-1}\\ & \;\cdot [{\bar{F}}_{n}-{\bar{G}}_{n}-{\hat{E}}_{n-1}\cdot K_{n-1}^{-1}\cdot {\bar{R}}_{n-1}\cdot {\hat{E}}_{n-1}\cdot ({\bar{F}}_{n}+{\bar{G}}_{n})]. \end{array}$$

### 4.3. Stabilized back-propagation

To compute ${\bar{a}}_{n}$ and **b**_*n*_ for equation (4.6), substitution of their reflection-matrix expressions from equations (4.10)–(4.11) into equations (4.12)–(4.13) yields an equation system with the solution

4.44 $$a_{n}=-\frac{i}{2}\mu_{n}(z_{s}){\left(I-{\bar{S}}_{n}\cdot S_{n}\right)}^{-1}\cdot \int_{J_{n}}\varphi (x_{s})[\Psi_{n}^{T}(x_{s},z_{s})+{\bar{S}}_{n}\cdot \Phi_{n}^{T}(x_{s},z_{s})]\;dx_{s}$$

and

4.45 $${\bar{b}}_{n}=-\frac{i}{2}\mu_{n}(z_{s}){\left(I-S_{n}\cdot {\bar{S}}_{n}\right)}^{-1}\cdot \int_{J_{n}}\varphi (x_{s})[\Phi_{n}^{T}(x_{s},z_{s})+S_{n}\cdot \Psi_{n}^{T}(x_{s},z_{s})]\;dx_{s},$$

where $S_{n}={\hat{E}}_{n}\cdot R_{n}$ and ${\bar{S}}_{n}={\hat{E}}_{n}\cdot {\bar{R}}_{n}$. This solution is of course completely analogous to the one in [46, eqns (39)–(40)]. Obvious simplifications, with vanishing terms, appear when *n* = 1 and when *n* = *N* + 1.

To compute the *y*-component displacement field *v*(*x*,*z*) according to equation (4.6), it remains to compute the column vectors ${\bar{a}}_{n}^{L}$, $b_{n}^{L}$ and ${\bar{a}}_{n}^{R}$, $b_{n}^{R}$ for *n* = 1, 2, …, *N* + 1. Considering the computation of the reflection matrices **R**_*n*_ and ${\bar{R}}_{n}$ as forward propagation steps, this is done by two passes of stabilized back-propagation. Starting from ${\bar{a}}_{1}^{L}=0$, successive matrix-vector multiplications according to the transmission equations (4.21), (4.26) and/or (4.29) yield ${\bar{a}}_{n}^{L}$ for increasing *n*. Analogously, starting from $b_{N+1}^{R}=0$, successive matrix-vector multiplications according to the transmission equations (4.34), (4.39) and/or (4.42) yield $b_{n}^{R}$ for decreasing *n*. Note that these computations involve decreasing exponentials by multiplications with ${\hat{E}}_{n}$ when $a_{n}^{L+}$ and ${\bar{b}}_{n}^{R+}$ are formed according to equations (4.18) and (4.31), respectively.

At the same time, to avoid multiplications with ${\hat{E}}_{n}^{-1}$ and spurious exponential magnification of round-off errors, $b_{n}^{L}$ and ${\bar{a}}_{n}^{R}$ are computed according to equations (4.10) and (4.11), respectively, using stabilization with the reflection matrices. In effect, the boundary conditions $b_{N+1}=b_{N+1}^{L}=0$ (**R**_*N*+1_ = **0**) and ${\bar{a}}_{1}={\bar{a}}_{1}^{R}=0$ (${\bar{R}}_{1}=0$), respectively, safely control these computations.

### 4.4. Example

**Figure 3.**
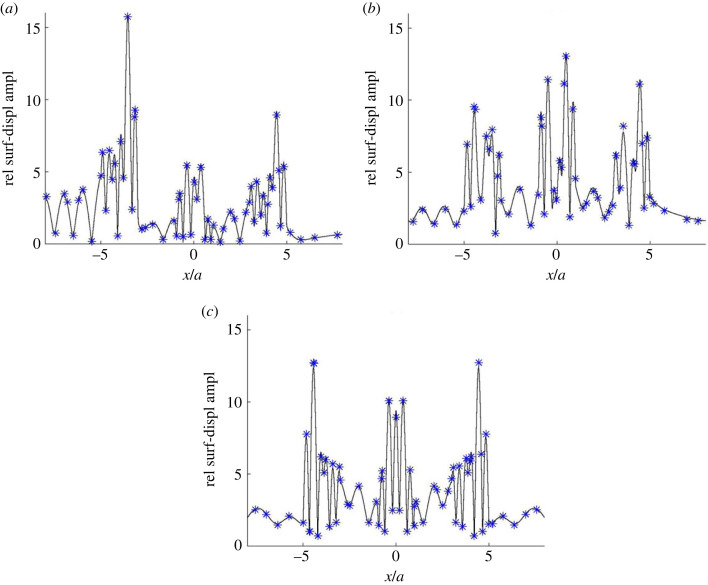
Coupled-mode surface-displacement amplitude curves for an example with three alluvium-valley anomalies as described in the text. A plane SH wave is incident from below at three different propagation angles relative to the positive *x*-axis: (*a*) 5°, (*b*) 45° and (*c*) 90°. The amplitude results are given relative to the incident-wave amplitude, and the star symbols indicate corresponding results from Zhang *et al.* [41, fig. 5].

Figure 3 concerns an example from Zhang *et al.* [41, Sec. 4.2]. The medium, without absorption and with a flat surface at *z* = 0 (km), is homogeneous except for three similar semicircular alluvial valleys with radius *a*, centred at (*x*,*z*) = (−4*a*,0), (0,0) and (4*a*,0), respectively. With *ρ* and *β* denoting alluvium density and shear-wave velocity, and *ρ*^0^ and *β*^0^ denoting corresponding values for the surrounding bedrock, *ρ*/*ρ*^0^ = 2/3 and *β*/*β*^0^ = 1/3. A plane SH wave with frequency *β*^0^/2*a* is incident from below at three different angles to the horizontal plane: (a) 5°, (b) 45° and (c) 90° (vertical incidence). In (a) and (b), the wave direction is to the right (increasing *x*).

For all numerical results, there are certain control parameters affecting the trade-off between accuracy and efficiency. The main ones for the present coupled-mode computations are the number of strip regions (*N* + 1), *z*_*b*_ and the other parameters for the artificial medium truncation, and the number of included normal modes in each strip region. For figure 3, each alluvial valley is discretized with about 70 strip regions of varying thickness. Compared to the wavelength (2*a* in the bedrock), a fine *x*-discretization is needed, adapted to the local alluvium–bedrock interface slope [47]. Furthermore, *z*_*b*_ = 50*a* for figure 3*a* and *z*_*b*_ = 15*a* for figure 3*b*,*c*. In each case, differential-evolution optimization according to §4.1, with *z*_*s*_ = 3*a*, provides suitable parameters for the artificial absorption at depth. The number of included normal modes in each strip region is 503 for figure 3*a* and 200 for figure 3*b*,*c*.

The surface-displacement amplitude results show satisfactory agreement with the corresponding ones (without absorption) in [41, fig. 5], indicated by star symbols in figure 3. Note the significant ground-motion amplification within the soft valleys. Also note the clear wave shielding of the middle valley by the left one in figure 3*a* with almost horizontal incidence from the left. The shielding of the right valley is less effective because of the slightly non-horizontal incidence, the greater distance from the left valley, and the shielding of the middle one. Using partial-wave decomposition, this example is revisited in §5.3 for quantitative assessment of (multiple) scattering by the alluvial valleys.

After the design of the artificial medium truncation, the computations involve the following four steps: computation of the modal horizontal wavenumbers *k*_*m*,*n*_, computation of the normalized mode functions *Z*_*m*,*n*_(*z*), computation of the mode-coupling matrices **F**_*n*_, **G**_*n*_ and ${\bar{F}}_{n}$, ${\bar{G}}_{n}$, and the actual coupled-mode computations with reflection-matrix recursion and stabilized back-propagation according to §§4.2 and 4.3. The first step is typically the most time-consuming. Note that the results from the first three steps can be reused for computations with other directions of plane-wave incidence, for example.

Assume, for simplicity, that the finite number of modes included in the computations, denoted *N*_*M*_, is about the same in all strip regions. In general, the needed *N* and *N*_*M*_ are both roughly proportional to the frequency. For the last of the four computation steps, the computational work is roughly proportional to $N\;N_{M}^{3}$, since the reflection-matrix recursions involve inversions of matrices of size *N*_*M*_ × *N*_*M*_. Some variations may of course occur, depending on the particular recursion option from §4.2 and the number of receivers. For figure 3, with a standard serial home personal computer, the corresponding CPU times are 481 s for figure 3*a*, and 27 s for each of figure 3*b*,*c*. The root-mean-square deviations from results obtained by doubling *N* and *N*_*M*_ are less than 1% of the maximum amplitude, indicating the accuracy.

## 5. Field decomposition into partial SH waves

The partial waves from §2 are now simply denoted *v*^0^ (the basic one), and $v_{\;j_{l},\ldots ,j_{2},j_{1}}^{\;j_{0}\sigma }$ (the additional ones). As before, σ=− or + and *j*_0_, *j*_1_, …, *j*_*l*_ denote anomaly regions (*A*, *B*, …). A $v_{\;j_{l},\ldots ,j_{2},j_{1}}^{\;j_{0}-}$ wave starts with a $b_{X=j_{0}}$ source vector to the left involving a Ψ function, while a $v_{\;j_{l},\ldots ,j_{2},j_{1}}^{\;j_{0}+}$ wave starts with an ${\bar{a}}_{X=j_{0}}$ source vector to the right involving a Φ function.

### 5.1. Reflection-matrix recursion with successive restarts

It is in fact easy to adapt a computer program for computation of the full field to computation of partial waves for specified anomaly regions and corresponding connection strips. Still apply the reflection-matrix recursions according to equations (4.23), (4.28), (4.30) and (4.36), (4.41), (4.43) throughout the whole medium, from *n* = *N* to *n* = 1 and from *n* = 2 to *n* = *N* + 1, respectively. Now, however, restart the recursion with a vanishing reflection matrix in the right-hand side upon entry to an anomaly region from one of the connection strips. This procedure automatically provides the elementary reflection matrices **R**_*A*_, **R**_*B*_, … and ${\bar{R}}_{A}$, ${\bar{R}}_{B}$, … for the anomaly regions, cf. figure 2.

It is not necessary to compute the transmission matrices **T**_*A*_, **T**_*B*_, … and ${\bar{T}}_{A}$, ${\bar{T}}_{B}$, … explicitly. Typically, transmission is best handled by sequential matrix-vector multiplications according to the technique with stabilized back-propagation from §4.3.

### 5.2. Computation of the additional partial waves

The transmission of a $v_{\;j_{l},\ldots ,j_{2},j_{1}}^{\;j_{0}\sigma }$ wave through a connection strip is easily done, using the appropriate ${\hat{E}}_{n}$ matrix. In particular, the pertinent $b_{X=j_{0}}$ or ${\bar{a}}_{X=j_{0}}$ vector is initially transmitted in this way. The transmission through the involved anomaly regions is done with stabilized back-propagation, incorporating the backward-going waves; cf. §4.3. The reflection matrices obtained by recursion with successive restarts according to §5.1 are the appropriate ones for this purpose, as well as for the reflections from the anomaly regions. Since the additional partial waves do not include source terms, except for the initial **b**_*X*_ or ${\bar{a}}_{X}$ vector, the involved equations (4.18) and (4.31) are simplified.

Concerning the reflections from the anomaly regions, it is instructive to consider a medium with two anomaly regions, *A* and *B*, separated by connection strip *n*. In strip *n*, $v(x,z)=v^{0}(x,z)+$ $\Phi_{n}(x,z)\cdot ({\bar{a}}_{A}+\Delta \bar{a})+\Psi_{n}(x,z)\cdot (b_{B}+\Delta b)$, where $\Delta \bar{a}={\bar{R}}_{n}\cdot {\hat{E}}_{n}\cdot (b_{B}+\Delta b)$ and $\Delta b=R_{n}\cdot {\hat{E}}_{n}\cdot ({\bar{a}}_{A}+\Delta \bar{a})$. It follows that

5.1 $$\Delta \bar{a}={\bar{R}}_{n}\cdot {\left(I-S_{n}\cdot {\bar{S}}_{n}\right)}^{-1}\cdot {\hat{E}}_{n}\cdot (R_{n}\cdot {\hat{E}}_{n}\cdot {\bar{a}}_{A}+b_{B})$$

and

5.2 $$\Delta b=R_{n}\cdot {\left(I-{\bar{S}}_{n}\cdot S_{n}\right)}^{-1}\cdot {\hat{E}}_{n}\cdot ({\bar{a}}_{A}+{\bar{R}}_{n}\cdot {\hat{E}}_{n}\cdot b_{B}).$$

Note the appearance of the same inverse matrices as in equations (4.44) and (4.45). Expansion of the inverse matrices in geometric series provides the additional partial waves. When *A* and/or *B* are split, with more anomaly regions and connection strips, the corresponding refined partial-wave decomposition follows from the two-port algebra according to §2.2 together with the elementary reflection matrices according to §5.1.

### 5.3. Narrow-band example

Returning to the example case from §4.4, introduce three anomaly regions: *A* for *x* < −3*a*, *B* for |*x*| < *a* and *C* for *x* > 3*a*. Note that each anomaly region covers one of the alluvium-valley anomalies. There are two intermediate connection strips: one for −3*a* < *x* < −*a*, and one for *a* < *x* < 3*a*. Restriction is now made to the case from figure 3*b*, with incident-wave propagation angle 45°.

**Figure 4.**
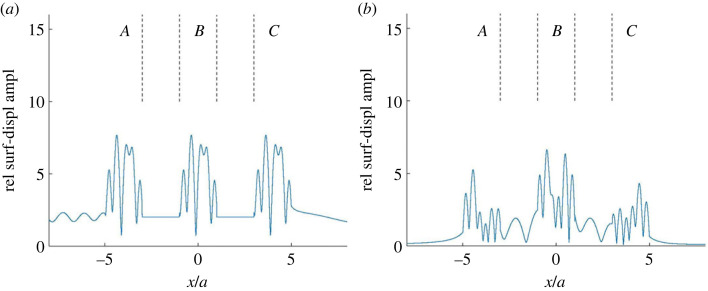
Further surface-displacement amplitude results for the case from figure 3 with incident-wave-propagation angle 45°: (*a*) the basic partial wave *v*^0^ and (*b*) the (coherent) sum of the most significant additional partial waves, 16 of which are shown in figure 5. The amplitude results are given relative to the incident-wave amplitude, and the anomaly regions (*A*, *B* and *C*) are indicated.

Figure 4*a* shows the corresponding basic partial wave *v*^0^. Note the constant relative amplitude 2 in the two connection strips, resulting from the reflection of the plane SH wave at the free surface. Some modulation of the relative amplitude 2 appears for *x* < −5*a* in anomaly region *A* and for *x* > 5*a* in anomaly region *C*, because of scattering from the alluvial valley in *A* and *C*, respectively. There is some asymmetry in the field because of the oblique incidence. As expected, the *v*^0^ fields within the three alluvial valleys agree.

**Figure 5.**
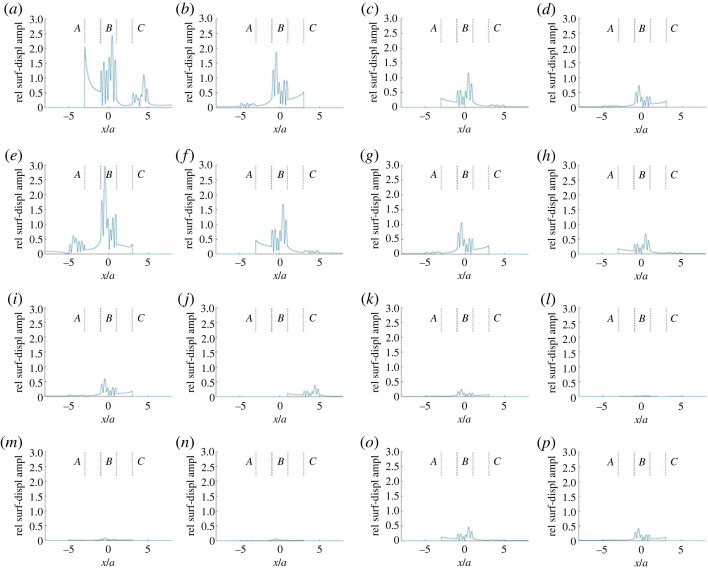
Some individual additional partial waves included in the sum in figure 4*b*. The first row shows the partial waves (*a*) *v*^*A*+^, (*b*) $v_{C}^{B+}$, (*c*) $v_{A,B}^{A+}$ and (*d*) $v_{C,B,C}^{B+}$. The second row shows (*e*) *v*^*C*−^, (*f*) $v_{A}^{B-}$, (*g*) $v_{C,B}^{C-}$ and (*h*) $v_{A,B,A}^{B-}$. The third row shows (*i*) $v_{C}^{A+}$, (*j*) $v_{B,C}^{A+}$, (*k*) $v_{C,B,C}^{A+}$ and (*l*) $v_{A,C}^{A+}$. The fourth row, finally, shows (*m*) $v_{C,A}^{B-}$, (*n*) $v_{C,A,B}^{A+}$, (*o*) $v_{A,B,A,B}^{A+}$ and (*p*) $v_{C,B,C,B}^{C-}$.

Figure 4*b* shows the (coherent) sum of all essential additional partial waves $v_{\;j_{l},\ldots ,j_{2},j_{1}}^{\;j_{0}\sigma }$. This field results from remaining effects of single and multiple scattering by the alluvial valleys. (As already noted, some single-scattering effects appear in figure 4*a*, for |*x*| > 5*a* there, because of the extension of anomaly regions *A* and *C* beyond their pertinent alluvial valleys.) In each anomaly region, particularly the middle one *B*, the scattering contributions from the other anomaly regions are significant. The scattering into the two connection strips is also essential. The (coherent) sum of the waves in figure 4*a*,*b* agrees very well with figure 3*b*.

As a complement to figure 4, with a different amplitude scale, figure 5 shows some individual additional partial waves $v_{\;j_{l},\ldots ,j_{2},j_{1}}^{\;j_{0}\sigma }$. Except *v*^*A*+^ and *v*^*C*−^, in figure 5*a*,*e*, respectively, all of them involve multiple scattering (reflections) by the alluvial valleys. Note the successively decreasing amplitudes in each of the two upper rows, because of the increasing number of reflections. Figure 5*k*–*n* shows, cf. $v_{A,B,A,B}^{A+}$ and $v_{C,B,C,B}^{C-}$ in figure 5*o*,*p* with five reflections, that transmission through anomaly regions may imply larger amplitude losses than reflections.

### 5.4. Broad-band example

For a broad-band example, consider [41, Sec. 5] with another case with a flat surface at *z* = 0 km and three similar semicircular alluvial valleys. This time, as illustrated in [41, fig. 9(b)], the valleys have radius 1 km and they are centred at (*x*,*z*) = ( − 8,0) km, (0,0) km, and (8,0) km, respectively. With *ρ* and *ρ*^0^ denoting the density in the alluvium and in the surrounding homogeneous bedrock, *ρ*/*ρ*^0^ = 2/3. The shear-wave velocities in the alluvium (with some absorption) and in the bedrock are 0.5 (1 − 0.1i)^1/2^ ≈ 0.501 − 0.025i km s^−1^ and 1 km s^−1^, respectively.

A plane SH wave is incident from below at the propagation angle 45° relative to the positive *x*-axis. The spectrum of the source pulse, not exactly as in [41, Sec. 5], is limited to the frequency band (0.07, 1.53) Hz. Coupled-mode computations for time-domain results are performed with Fourier synthesis using about 50 discrete frequencies within this band. Each alluvial valley is thereby discretized with about 35 strip regions, for simplicity the same number for all frequencies. The source-array depth *z*_*s*_ is 2 km, while the depth *z*_*b*_ varies from 5 km for the highest frequencies to 21 km for the lowest ones. As in the previous example, differential-evolution optimization according to §4.1 furnishes appropriate parameters for the artificial absorption. In this broad-band case, good results are obtained with only 45 normal modes in each strip region, for each frequency. With averaging over frequencies, fewer modes are apparently needed than for narrow-band cases.

**Figure 6.**
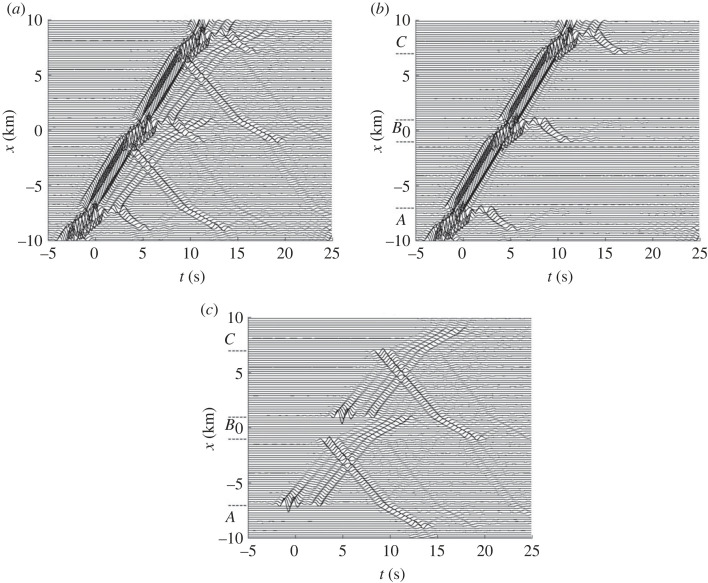
Coupled-mode surface-displacement time traces for an example with three alluvium-valley anomalies as described in the text. A plane SH wave is incident from below at the propagation angle 45° relative to the positive *x*-axis. The three panels show: (*a*) total result, (*b*) the basic partial wave *v*^0^ and (*c*) the (coherent) sum of the most significant additional partial waves, six of which are shown in figure 7. The anomaly regions (*A*, *B* and *C*) are indicated in (*b*,*c*).

Figure 6*a* shows 100 time traces for the surface displacements between *x* = −10 km and *x* = 10 km and a certain time window of length 30 s. The arrivals within |*x*| < 4 km can be favourably compared to those in [41, fig. 10(e)].

**Figure 7.**
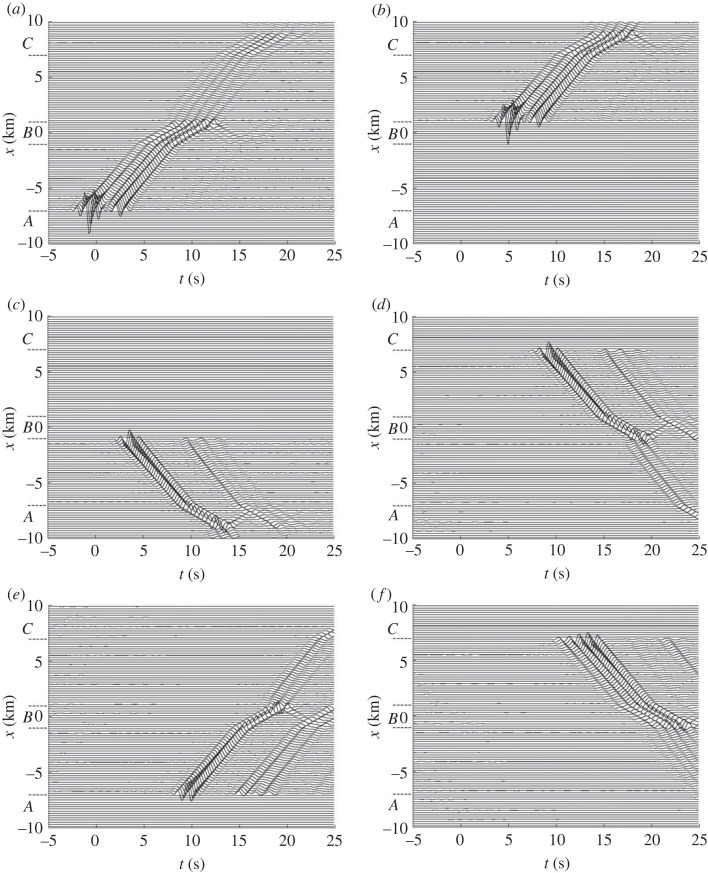
Some individual additional partial waves included in the sum in figure 6*c*. The first and second rows show the partial waves (*a*) *v*^*A*+^, (*b*) *v*^*B*+^, (*c*) *v*^*B*−^ and (*d*) *v*^*C*−^. These waves are magnified three times in relation to those in figure 6. The third row shows (*e*) $v_{A}^{B-}$ and (*f*) $v_{C}^{B+}$, magnified 20 times in relation to the waves in figure 6.

To aid the interpretation of the arrivals, using partial waves, introduce three anomaly regions: *A* for *x* < −7 km, *B* for |*x*| < 1 km and *C* for *x* > 7 km. Note that each anomaly region covers one of the alluvium-valley anomalies. There are two intermediate connection strips: one for −7 km < *x* < −1 km, and one for 1 km < *x* < 7 km.

Figure 6*b* shows the corresponding basic partial wave *v*^0^. Within the connection strips, there is of course only one arrival, the direct one as doubled by the surface reflection. Within an alluvial valley, there is a delayed direct arrival followed by a later reflection from the semicircular valley boundary; cf. [41, fig. 10(b)] for |*x*| < 1 km. Of course, the *v*^0^ fields within the three alluvial valleys agree. Because of the extension of anomaly regions *A* and *C* beyond their pertinent alluvial valleys, there is a reflected arrival from the valley in *A* for *x* < −9 km, and a scattered arrival from the valley in *C* for *x* > 9 km. For the middle valley, the corresponding arrivals are clearly seen in the total field of figure 6*a*. In [41, fig. 10(b,e)], they are denoted SL01 and SR01, respectively.

Figure 6*c* shows the (coherent) sum of all essential additional partial waves $v_{\;j_{l},\ldots ,j_{2},j_{1}}^{\;j_{0}\sigma }$. The (coherent) sum of the waves in figure 6*b*,*c* agrees very well with figure 6*a*. Note the rather strong waves at (*x*, *t*) ≈ (−6.9 km, −1 s) and (*x*, *t*) ≈ (1.1 km, 4.5 s) in figure 6*c*, caused by scattering by the right ends of the left and middle alluvial valleys, respectively. By destructive interference with the corresponding displacements in figure 6*b*, some slight wave shielding appears in figure 6*a*. This wave shielding is of course more significant at more horizontal incidence [41, Sec. 5].

Magnified compared to the previous figure, figure 7 shows some individual additional partial waves $v_{\;j_{l},\ldots ,j_{2},j_{1}}^{\;j_{0}\sigma }$. It is clear that the rather strong waves at (*x*, *t*) ≈ (−6.9 km, −1 s) and (*x*, *t*) ≈ (1.1 km, 4.5 s) in figure 6*c* belong to *v*^*A*+^ shown in figure 7*a* and *v*^*B*+^ shown in figure 7*b*, respectively. Reflections from the front as well as back sides of an alluvial valley give rise to a clear doublet structure of the partial waves in figure 7*c*–*f*. A doublet structure, albeit weak, can be discerned in figure 7*a*,*b* too. It is caused by reflections back and forth within anomaly regions *A* and *B*, respectively. Forward scattering from the alluvial valley within anomaly region *j*_0_ causes prolongation of the initial arrival for $v_{\;j_{l},\ldots ,j_{2},j_{1}}^{\;j_{0}+}$. This is clearly seen for *v*^*A*+^, *v*^*B*+^ and $v_{C}^{B+}$ in figure 7.

Within a traversed alluvial valley, reflections from its far side appear for each of the additional partial waves in figure 7. Note that the multiply scattered (reflected) waves $v_{A}^{B-}$ and $v_{C}^{B+}$, significantly magnified in figure 7*e*,*f*, are weak and barely notable in figure 6*c*. Effects of multiple scattering can be larger when the anomalies are closer together.

## 6. SH waves in periodic media

Assume that *N* ≥ 2 and modify the *y*-independent solid medium from §4 for *x* < *x*_1_ and *x* > *x*_*N*_, such that the resulting medium is periodic with period *d* = *x*_*N*_ − *x*_1_. In particular, *z*_*a*;1_ = *z*_*a*;*N*_, *ρ*_1_(*z*) = *ρ*_*N*_(*z*), *β*_1_(*z*) = *β*_*N*_(*z*), *z*_*a*;2_ = *z*_*a*;*N*+1_, *ρ*_2_(*z*) = *ρ*_*N*+1_(*z*) and *β*_2_(*z*) = *β*_*N*+1_(*z*). Furthermore, the source function φ(*x*) satisfies φ(*x* + *d*) = φ(*x*).

**Figure 8.**
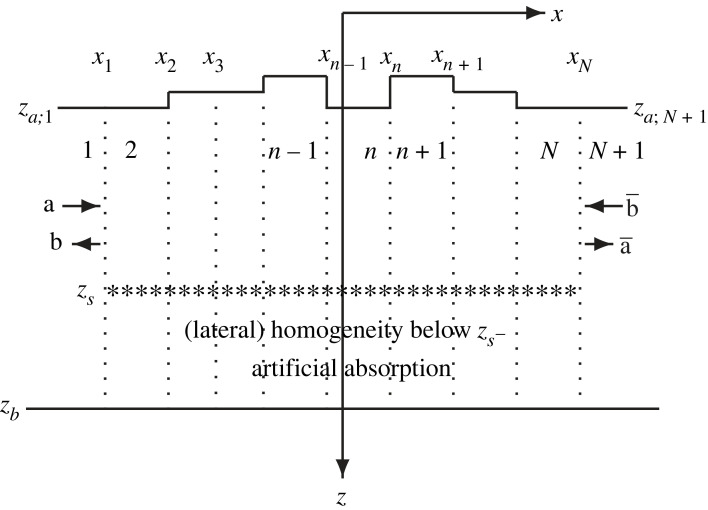
Vertical *xz*-plane as in figure 1*b*, but for the periodic medium with unit cell between *x*_1_ and *x*_*N*_. The vectors **a** and $\bar{b}$ provide input from the surrounding strip regions, with corresponding output vectors **b** and $\bar{a}$.

For computation of the full field, the aim is now to reduce the computations to a single period or unit cell: the one between *x*_1_ and *x*_*N*_. To that end, regard this part of the medium as a two-port with input field vectors **a** and $\bar{b}$ from the strip regions to the left and right, respectively, and corresponding output field vectors **b** and $\bar{a}$ to these strip regions, respectively. Figure 8 gives an illustration.

### 6.1. Computations for the unit cell with one medium period

A difference from the two-port discussion in §2.2 is that the sources at *z* = *z*_*s*_ within the two-port now contribute. In the present case, cf. equation (2.1),

6.1 $$b=R\cdot a+\bar{T}\cdot \bar{b}+b_{S}\;\mathrm{and}\;\bar{a}=T\cdot a+\bar{R}\cdot \bar{b}+{\bar{a}}_{S}$$

with **b**_*S*_ and ${\bar{a}}_{S}$ representing the contributions from the sources. These source vectors are readily computed according to §4 for the related non-periodic medium with strip regions 1 and *N* + 1 extending to *x* = −∞ and *x* = ∞, respectively, and with the source function φ(*x*) set to zero outside the unit cell.

Assume, for simplicity, that the periodic medium is laterally continuous across *x* = *x*_1_ and *x* = *x*_*N*_, such that *z*_*a*;1_ = *z*_*a*;2_ = *z*_*a*;*N*_ = *z*_*a*;*N*+1_, *ρ*_1_(*z*) = *ρ*_2_(*z*) = *ρ*_*N*_(*z*) = *ρ*_*N*+1_(*z*) and *β*_1_(*z*) = *β*_2_(*z*) = *β*_*N*_(*z*) = *β*_*N*+1_(*z*), and that φ(*x*) is a regular function at *x* = *x*_1_ and *x* = *x*_*N*_. Then it follows by periodicity that

6.2 $$\bar{a}=E\cdot a\;\mathrm{and}\;\bar{b}=E\cdot b,$$

where **E** = diag_*m*_ (exp(i*k*_*x*_*d*)) = exp(i*k*_*x*_*d*) **I**. Solution of equations (6.1) and (6.2) yields

6.3 $$b={\left[I-(\bar{T}+R\cdot {\left(E-T\right)}^{-1}\cdot \bar{R})\cdot E\right]}^{-1}\cdot [R\cdot {\left(E-T\right)}^{-1}\cdot {\bar{a}}_{S}+b_{S}]$$

and

6.4 $$a={\left[E-T-\bar{R}\cdot E\cdot {\left(I-\bar{T}\cdot E\right)}^{-1}\cdot R\right]}^{-1}\cdot [\bar{R}\cdot E\cdot {\left(I-\bar{T}\cdot E\right)}^{-1}\cdot b_{S}+{\bar{a}}_{S}].$$

The field in the unit cell follows by summing the solution for the related non-periodic medium and the transmitted fields arising from the vectors **a** from the left and $\bar{b}$ from the right, respectively. These transmitted fields are efficiently computed by stabilized back-propagation, this time using the reflection matrices available from the handling of the related non-periodic medium.

Explicit computation of the transmission matrices **T** and $\bar{T}$ is actually needed in this case, to compute **b** and **a** from equations (6.3) and (6.4). Concerning **T**, matrices from equations (4.21), (4.26) and (4.29) must be multiplied, while $\bar{T}$ involves matrices from equations (4.34), (4.39) and (4.42).

### 6.2. Example

**Figure 9.**
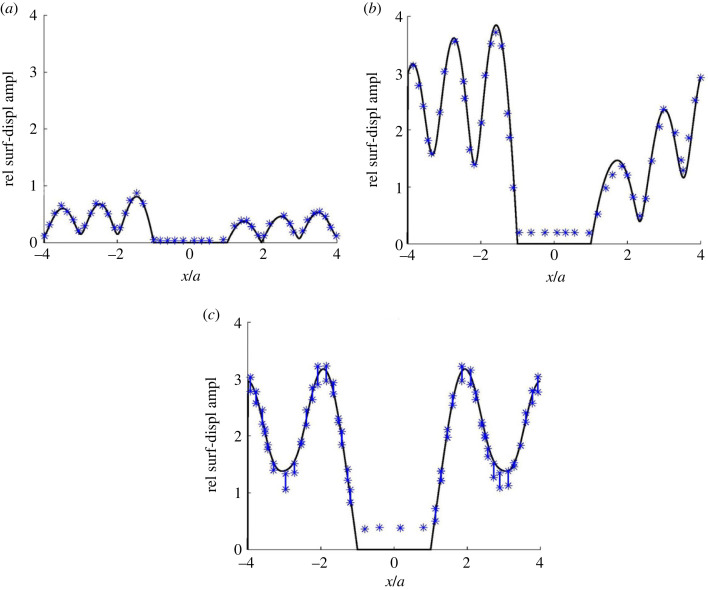
Coupled-mode surface-displacement amplitude curves for the example with peridically distributed semicircular rigid boundaries at |*x* − 8*la*| < *a* for *l* = 0, ±1, ±2, …. A plane SH wave is incident from below at three different propagation angles to the positive *x*-axis: (*a*) 5°, (*b*) 45° and (*c*) 90°. The amplitude results are given relative to the incident-wave amplitude, and the star symbols indicate corresponding results from Zhang *et al.* [41] and Ba & Liang [56]. The Zhang *et al.* [41] and Ba & Liang [56] results are often somewhat different for the 90° angle. At each *x*, the two star symbols in (*c*) are, therefore, connected by a vertical line segment for visual clarity.

Figure 9 concerns an example from Zhang *et al.* [41, Sec. 3.2], originally treated in [56, Sec. 5.2]. The medium, without absorption, is now homogeneous with a flat and free surface at *z* = 0 (km) interrupted by an infinite number of periodically distributed down-going semicircular rigid (!) boundaries with radius *a*, centred at (*x*,*z*) = (8*la*,0) for *l* = 0, ±1, ±2, …. As in §4.4, a plane SH wave with frequency *β*^0^/2*a*, where *β*^0^ is the shear-wave velocity, is incident from below at three different angles to the horizontal plane: (a) 5°, (b) 45° and (c) 90° (vertical incidence). In (a) and (b), the wave direction is to the right (increasing *x*).

So far, the upper (solid-)medium boundary has been assumed to be free. As detailed in appendix A, the rigid case necessitates some modifications of §§4.2 and 3.

The coupled-mode computations for figure 9 are restricted to the unit cell with |*x*| < 4*a*, and the involved semicircular anomaly at |*x*| < *a* is discretized with about 70 strip regions of varying thickness; cf. the *x*-discretization in §4.4. The depths *z*_*b*_ and *z*_*s*_, as well as the parameters for artificial absorption, are as in §4.4, for each of the three incidence-angle cases.

The surface-displacement amplitude results in figure 9 agree well with the corresponding ones in [41, fig. 3] and [56, fig. 9], which are indicated by star symbols in figure 9. Note the vanishing coupled-mode displacements at the semicircular boundary. The results in the two previous papers were obtained using high-velocity semicircular inclusions to mimic the desired rigid case, leading to non-vanishing corresponding displacements.

Using the technique with partial waves from §§2 and 5, it would be easy to investigate how the response in figure 9 arises from multiple scattering among the down-going semicircular rigid-boundary anomalies. Result curves of the same type as in figures 4 and 5 would appear.

## 7. Computational variants

The present paper focuses on modal reflection matrices as a convenient tool to transport boundary conditions along the *x*-axis: **R** = **0** from the right end, and $\bar{R}=0$ from the left end. These reflection matrices relate coefficient column vectors for an expansion of *v*(*x*, *z*) in each strip *n* in terms of the row vectors $\Phi_{n}(x,z)$ and $\Psi_{n}(x,z)$; cf. equations (4.6), (4.10) and (4.11).

By the definition of $\Phi_{n}(x,z)$ and $\Psi_{n}(x,z)$, expansions of *μ*_*n*_(*z*) ∂*v*(*x*,*z*)/∂*x* and −i*ωv*(*x*,*z*) in each strip *n* in terms of the corresponding row vector **Z**_*n*_(*z*) = {*Z*_*m*,*n*_(*z*)} follow from the previous expansion of *v*(*x*, *z*). Linear relations between the corresponding coefficient column vectors appear with so-called impedance and admittance matrices, which are easy to relate to the reflection matrices; cf. [57, eqn (13)]. Obviously, related recursions for impedance matrices could be used instead of the reflection-matrix recursions in §4.2, and the boundary conditions could be transported using impedance matrices. Some texts, e.g. [57] for shape optimization of acoustic horns and [58] for acoustic simulation of the vocal tract, apply the impedance-matrix approach. Just as for the reflection matrices [7], differential equations of Riccati type appear in the continuous case without medium discretization.

It is also natural to compare to well-known methods for computation of seismic P-SV waves in multilayered laterally homogeneous media. After Fourier- or Hankel-transformation of a horizontal coordinate to the wavenumber domain, a two-point boundary-value problem appears for a system of ordinary differential equations in the depth variable *z*. Several numerical methods have been proposed to solve this problem in an unconditionally stable way. For example, Kennett [16] combines 2 × 2 *R*/*T* matrices for the different layers recursively, while Wang & Rokhlin [59] compute a 4 × 4 global stiffness matrix recursively from individual layer stiffness matrices. Stiffness and compliance matrices relate stresses to displacements, and vice versa, combining *both* sides of a (composite) layer. Concerning *v*(*x*, *z*) in the present paper, a corresponding stiffness-matrix method would obviously relate the two expansion column vectors for *μ*_*n*_(*z*) ∂*v*(*x*,*z*)/∂*x* at two different *x*-values, with corresponding *n*-values, to the two expansion column vectors for *v*(*x*, *z*) at these two *x*-values. Equation (2.1) would be useful to relate stiffness and compliance matrices to *R*/*T* matrices, but these things are not pursued here.

To avoid spurious reflections from down-going waves, an artificial medium truncation involving a classical absorbing layer is carefully designed using global optimization in §4.1. It is a convenient choice, since standard methods for mode expansion with computation of modal wavenumbers and mode functions are directly applicable. There are good alternatives, however, which should be able to remove the spurious reflections with a much thinner artificial layer. Givoli [60] describes some milestones in the development of absorbing boundaries and layers, including Dirichlet-to-Neumann boundary conditions, PML (perfectly matched layer), and high-order absorbing boundary conditions. The main interest has concerned applications for purely numerical methods, such as finite elements and finite differences. This is true for the PML approach too, with a recent review in [61], but this technique has attracted some interest within a modal framework as well.

With PML, so-called PML modes appear in addition to trapped and leaky modes [62]. These PML modes are significant mainly within the PML region. The inclusion of PML modes in the modal basis may need some care, however. For a Pekeris waveguide, Zhu & Lu [63] provide approximate solutions for the PML modes, which replace the pertinent branch-cut integral. According to Zhu & Zhang [64], the eigenfunctions of the modified Helmholtz operator have no orthogonality in a bounded domain with a PML, and the authors derive pertinent conjugate eigenfunctions for the case of a Pekeris waveguide.

## 8. Concluding remarks

For a solid medium that is invariant in the horizontal *y*-coordinate direction, §2 presents a mathematically exact decomposition of the seismic wavefield with partial waves. With §§3 and 4 as additional background, details and examples for the scalar case with pure SH waves follow in §5. The decomposition is defined using discrete coupled-mode theory and combination of elementary reflection matrices, conveniently computed by recursion with successive restarts. It facilitates physical interpretation and allows detailed assessment of multiple scattering among horizontally displaced anomaly regions. Related field decompositions into partial waves have been briefly indicated at the ends of [55, Sec. V B] and [46, Sec. VI]. The emphasis there is on reflections (or scattering) from the interiors and exteriors, or sides, of particular source and receiver regions.

Essentially as an adaptation of the 3D point-source case in [46, Sec. V], §4 develops the details of a discrete coupled-mode computation method for 2D SH-wave scattering at plane-wave incidence from below. The medium is discretized into a number of laterally homogeneous strip regions separated by vertical interfaces. A horizontal source array generates the incident plane SH wave according to equation (3.3) in §3. There is an artificial boundary at depth *z*_*b*_, allowing a normal-mode representation of the field in each strip region. Global optimization techniques are applied to design artificial absorption in a layer above this boundary to minimize reflections from it (§4.1).

Recursion of modal reflection matrices and stabilized back-propagation of modal expansion-coefficient vectors are essential features of the computation method. Compared to the coupled-mode method for a 3D point source in [46, Sec. V], the introduction of a horizontal source array necessitates a double pass of the stabilized back-propagation: a full pass in each direction (§4.3), to pick up source contributions from the left and from the right, respectively. To add a lot of point- or line-source contributions, with stabilized back-propagation in both directions from each, would not be efficient. As a consequence, the ${\bar{a}}_{n_{s}}$ and $b_{n_{s}}$ vectors from Ivansson [46, eqn (35)] are split in equation (4.6) into ${\bar{a}}_{n}$, ${\bar{a}}_{n}^{L}$, ${\bar{a}}_{n}^{R}$ and **b**_*n*_, $b_{n}^{L}$, $b_{n}^{R}$, respectively. The source distribution within each *x*-segment is handled analytically by integration. Modifications to handle a periodic medium efficiently appear in §6.

For the related continuous coupled-mode method of Kennett [7], the stabilized back-propagation would correspond to integration of equation (3.1) there as modified by insertion of equation (3.4) there. Note that the stabilized back-propagation avoids explicit computation of transmission matrices by matrix multiplications. Transmission through a sequence of strip regions, with corresponding field computations, only involves sequential matrix-vector multiplications according to equations (4.21), (4.26), (4.29), (4.34), (4.39), (4.42), (4.10) and (4.11). Composite transmission matrices are only needed in §6, for computation of the full field in a periodic medium with restriction to a single unit cell.

Note that the plane wave according to equation (3.3) breaks down if *k*_*x*_ = *ω*/*β*. To handle a horizontally incident plane wave, either make an approximation with a slightly sloping incidence, or use a point source or vertical source array, cf. [54, Sec. 3.3.3], at a far range. A comparatively large *z*_*b*_ may be needed (§4.1).

The illustrative examples (§§4.4, 5.3, 5.4 and 6.2) are all taken from Zhang *et al.* [41], focusing on multiple semicircular anomalies. Compared to the (semi-)analytical method in [41], the presented coupled-mode approach allows direct application of addition rules for *R*/*T* matrices to isolate partial waves and handling of anomalies of arbitrary shape, for computation of the total field as well as partial waves. Applications to (multiple) canyons, basins, tunnels, layered inclusions, etc., and combinations thereof, are straightforward. To handle a cavity in the 2D medium, use strip regions with two parts, one above and one below the cavity. Calculate modes separately for each of two such parts. Let a mode for an upper part vanish in the corresponding lower part, and vice versa. The method is particularly convenient for a medium with rectangular anomalies, without any need for discretization of a sloping boundary.

Except for §2, only the pure SH case is treated in the present paper. Extension to the case with a *y*-coordinate dependence of the waves, according to a factor exp(i*k*_*y*_*y*), would be possible, however. Conversions between SH (Love) and P-SV (Rayleigh) modes would appear at the vertical *x* = *x*_*n*_ interfaces. In equation (4.6), *v* would be replaced by the displacement vector **u** = (*u*, *v*, *w*)^T^, with components *u*, *v*, *w* in the *x*-, *y*-, *z*-directions, respectively. Correspondingly, $\Phi_{n}$ and $\Psi_{n}$ would be matrices with three rows, and the *Z*_*m*,*n*_ would be column vectors **Z**_*m*,*n*_. As shown in [9], appropriate orthogonality relations exist for the modes. Actually, the reflection-matrix formalism in §§4.2 and 4.3 would be applicable with minor changes, but the mode-coupling matrices would of course be different. They would involve Rayleigh–Rayleigh, Love–Love, as well as Rayleigh–Love coupling.

## Data Availability

The author’s computer programs are based on and include codes which can be downloaded from OALIB (Ocean Acoustics Library) at https://oalib-acoustics.org/. These codes are within the RPRESS package for Wavenumber Integration there. Needed additional codes appear as electronic supplementary material [65]. Scripts for using the computer programs to generate the author’s computational results in the figures also appear as electronic supplementary material [65].

## Appendix A. Modification of §4.2 for a different type of upper boundary

When part of the upper solid-medium boundary is rigid rather than free, some modifications are needed. This is relevant for the example in §6.2. Of course, the computation of the modal horizontal wavenumbers *k*_*m*,*n*_ and normalized mode functions *Z*_*m*,*n*_ must respect the free or rigid-boundary condition at *z*_*a*;*n*_ for each strip region *n*.

Concerning §4.2, the integrations over the pertinent depth intervals must be reconsidered; cf. the difference between Secs IV and III in [46]. In §4.2.1, equations (4.19)–(4.23) and (4.24)–(4.28) now apply when *I*_*n*+1_⊆ *I*_*n*_ and $I_{n+1}⊇I_{n}$, respectively. In §4.2.2, equations (4.32)–(4.36) and equations (4.37)–(4.41) now apply when *I*_*n*−1_⊆ *I*_*n*_ and $I_{n-1}⊇I_{n}$, respectively. Of course, these interchanges of the equations also apply when they are used in connection with the stabilized back-propagation according to §4.3.

## Appendix B. Addition to Ivansson [46, Sec. V]

Note that, after division with the reference length *r*_ref_, the integrand $\hat{\;p}(x,z;\kappa )\cos(\kappa (y-y_{s}))$ in [46, eqn (29)] solves the physical line-source problem with body force per unit volume given by −(*M*/2*πr*_ref_) grad[*δ*(*x* − *x*_*s*_) cos(*κ*(*y* − *y*_*s*_)) *δ*(*z* − *z*_*s*_)]. The inclusion of the ‘normalization factor’ $\pi^{-1}{\left(k_{m,n}^{2}-\kappa^{2}\right)}^{-1/2}$ in [46, eqn (30)], implying a variable transformation for the appearing column vectors **a**_*n*_ and ${\bar{a}}_{n_{s}}$, necessitates addition of the following paragraph at the end of Ivansson [46, Sec. V A]. It was unfortunately forgotten there. Some of the resulting equations are similar to corresponding ones in §4.2 of the present paper.

Finally, the normalization factor in eqn (30) necessitates some additional changes. With $K_{n}={\mathrm{diag}}_{m}(\pi {\left(k_{m,n}^{2}-\kappa^{2}\right)}^{1/2})$, replace **A**_*n*_ with $K_{n+1}\cdot A_{n}\cdot K_{n}^{-1}$, **B**_*n*_ with **K**_*n*+1_ · **B**_*n*_, and **C**_*n*_ with $C_{n}\cdot K_{n}^{-1}$. Furthermore, replace ${\bar{B}}_{n}$ with ${\bar{B}}_{n}\cdot K_{n}^{-1}$, ${\bar{C}}_{n}$ with $K_{n-1}\cdot {\bar{C}}_{n}$, and ${\bar{D}}_{n}$ with $K_{n-1}\cdot {\bar{D}}_{n}\cdot K_{n}^{-1}$. In eqns (9) and (13), replace **a**_*n*+1_ with $K_{n+1}^{-1}\cdot a_{n+1}$ and **a**_*n*_ with $K_{n}^{-1}\cdot a_{n}$. In eqns (10)–(11) and (14)–(15), replace **R**_*n*+1_ with **R**_*n*+1_ · **K**_*n*+1_ and **R**_*n*_ with **R**_*n*_ · **K**_*n*_. In eqns (18)–(19) and (22)–(23), replace ${\bar{R}}_{n-1}$ with $K_{n-1}^{-1}\cdot {\bar{R}}_{n-1}$ and ${\bar{R}}_{n}$ with $K_{n}^{-1}\cdot {\bar{R}}_{n}$.
